# Automated model calibration with parallel MCMC: Applications for a cardiovascular system model

**DOI:** 10.3389/fphys.2022.1018134

**Published:** 2022-11-09

**Authors:** Finbar Argus, Debbie Zhao, Thiranja P. Babarenda Gamage, Martyn P. Nash, Gonzalo D. Maso Talou

**Affiliations:** ^1^ Auckland Bioengineering Institute, University of Auckland, Auckland, New Zealand; ^2^ Department of Engineering Science, University of Auckland, Auckland, New Zealand

**Keywords:** MCMC, parameter identification, cerebral pressure estimation, identifiability, core predictions, uncertainty quantification

## Abstract

Computational physiological models continue to increase in complexity, however, the task of efficiently calibrating the model to available clinical data remains a significant challenge. One part of this challenge is associated with long calibration times, which present a barrier for the routine application of model-based prediction in clinical practice. Another aspect of this challenge is the limited available data for the unique calibration of complex models. Therefore, to calibrate a patient-specific model, it may be beneficial to verify that task-specific model predictions have acceptable uncertainty, rather than requiring all parameters to be uniquely identified. We have developed a pipeline that reduces the set of fitting parameters to make them structurally identifiable and to improve the efficiency of a subsequent Markov Chain Monte Carlo (MCMC) analysis. MCMC was used to find the optimal parameter values and to determine the confidence interval of a task-specific prediction. This approach was demonstrated on numerical experiments where a lumped parameter model of the cardiovascular system was calibrated to brachial artery cuff pressure, echocardiogram volume measurements, and synthetic cerebral blood flow data that approximates what can be obtained from 4D-flow MRI data. This pipeline provides a cerebral arterial pressure prediction that may be useful for determining the risk of hemorrhagic stroke. For a set of three patients, this pipeline successfully reduced the parameter set of a cardiovascular system model from 12 parameters to 8–10 structurally identifiable parameters. This enabled a significant (>4×) efficiency improvement in determining confidence intervals on predictions of pressure compared to performing a naive MCMC analysis with the full parameter set. This demonstrates the potential that the proposed pipeline has in helping address one of the key challenges preventing clinical application of such models. Additionally, for each patient, the MCMC approach yielded a 95% confidence interval on systolic blood pressure prediction in the middle cerebral artery smaller than ±10 mmHg (±1.3 kPa). The proposed pipeline exploits available high-performance computing parallelism to allow straightforward automation for general models and arbitrary data sets, enabling automated calibration of a parameter set that is specific to the available clinical data with minimal user interaction.

## 1 Introduction

Projects such as the Virtual Physiological Human initiative (Hunter et al., 2010) and the Physiome Project (Hunter et al., 2005) aim to create integrated whole-body models of human physiology that account for cellular, tissue, organ, and system level mechanisms. For these models to be clinically viable, they need to be efficiently calibrated to patient data. This calibration enables the creation of patient-specific models that can be used at the bedside. These models have the potential to aid in diagnosis and prognosis, and to optimise treatment strategies.

Calibration of models to patient data involves the optimisation of a large number of parameters, which is impractical as a manual task. Moreover, state-of-the-art automatic calibration techniques require a number of model executions that scales poorly with the number of model parameters. Thus, to perform this calibration task, parallel model executions and a large number of computational resources are required. This paper aims to create an automatic pipeline for identifying model parameters with parallelised methods suitable for high-performance computing (HPC). As a first use case, we demonstrate this pipeline on an *in silico* lumped parameter cardiovascular system model. This case was selected as it can be extended to include coupling of multiple organs in terms of oxygen and metabolic demands to help start to develop integrated models of human physiology.

Cardiovascular system (CVS) modelling has been developing for decades with a steady increase in complexity, but more work is required to ensure models can be calibrated to clinically available data. Ursino and coworkers (Ursino, 1998; Ursino and Magosso, 2000; Ursino and Magosso, 2003; Albanese et al., 2016) created pioneering lumped parameter models of the CVS. These models required various assumptions for parameters in the heart, circulatory system, and pulmonary system to produce a qualitatively accurate model. Blanco and coworkers developed various anatomically detailed models of the CVS that solved one-dimensional Navier-Stokes equations for the vessel flow dynamics (Blanco and Feijóo, 2013; Blanco et al., 2014; Blanco et al., 2015). Blanco et al. also showed that although simplified models of the CVS can be sufficient for the study of systemic indices of the overall circulation, more detailed models are required to analyse intricate circulatory system dynamics, particularly in the case of disease. Safaei et al. (2018) developed a 218 compartment closed-loop model of the circulatory system using a similar bond-graph (BG) approach as the one detailed in the present work. However, model calibration was performed manually or semi-automatically *via* ad hoc methodologies in all of these studies.

When calibrating a model, it is important to verify that the model has not been overfitted to the available data, and therefore, can be used for prediction of unobserved variables (For a reader accustomed to control theory, the “prediction” mentioned in this work is equivalent to unobserved state estimation). This work discusses structural identifiability, practical identifiability, and core predictions, three different but related approaches for ensuring a calibrated model is reliable. Structural and practical identifiability (Raue et al., 2009; Miao et al., 2011) are focused on ensuring the identified parameter set is unique. In turn, core predictions (Cedersund and Roll, 2009; Cedersund, 2012) focuses on the uncertainty bound of the model’s task-specific predictions. In the current literature, it is a less well explored approach but heavily inspires this work.

A model is structurally identifiable with respect to the measured data if it has one unique optimal parameter set. Specifically, a parameter is structurally unidentifiable if changing the parameter does not necessarily alter the discrepancy between the model output and the measurements because the change in the parameter can be compensated by modifying other parameters (Wieland et al., 2021). The model is structurally unidentifiable if it has any structurally unidentifiable parameters. Importantly, structural identifiability is only dependent on the structure of the equations and which states are measured, and does not depend on the value or the noise of the measurements. Practical identifiability, has multiple definitions. The definition used by Raue et al. (2009) can be briefly explained by explaining what it means for a parameter to be practically unidentifiable. It means that there is a manifold in the parameter space where the parameter posterior distribution is constant, so a finite confidence interval does not exist. As in the structural case, a model is only practically identifiable if all of its parameters are practically identifiable. In the case of practical identifiability, the parameter posterior distribution depends on the measurement data uncertainty as well as the model structure.

A core prediction is a uniquely identified model property that must be fulfilled for the model structure to explain the data (Cedersund, 2012). Often, this core prediction can be a model output which is crucial to the intended use of the model and its associated uncertainty. For a core prediction to be satisfied, the uncertainties of the calibrated parameters must enable a unique prediction to be made with a finite confidence interval. In this work, as described in the work by Cedersund and Roll (2009), Cedersund (2012), for the calibration process to be acceptable, we additionally require that the core prediction uncertainty is within an acceptable bound. This extension allows a researcher or clinician to take into account external factors when deciding if a prediction uncertainty is acceptable. For example, an acceptable uncertainty may depend on where the minimum and maximum of the confidence intervals lie with respect to clinical thresholds for treatment indications, the risk of possible treatments, the risk of performing an invasive measurement, and other factors that depend on the state of the patient. Importantly, it may also depend on whether the uncertainty is too large to confirm a diagnosis or disease classification. Using core predictions to verify that a model has been adequately calibrated for the scenario of interest has the advantage of not necessarily requiring uniquely identified parameters (Eydgahi et al., 2013). Therefore, it reduces the constraints on the parameter identification process, making it easier to calibrate a model that can provide task-specific predictions.

The complexity of multi-scale physiological models can create highly nonlinear relationships between model parameters and the desired outputs, which presents challenges for the analysis of global identifiability and model calibration. It is especially difficult to calibrate complex models to data in a way that allows general use of the model. This is the aim of practical identifiability approaches that focus on ensuring that the cost function is sensitive to every parameter in all regions of parameter space (Raue et al., 2009; Simpson et al., 2020). In contrast, the current work follows the idea that to ease the fitting of parameters, a model should be calibrated in a way that is specific to the desired task of the model (Villaverde and Banga, 2014). Therefore, if the uncertainty of the task-specific prediction is within user-defined bounds, the model is deemed to be acceptably calibrated.

Many approaches have been developed that can be used for the calculation of task-specific predictions and their uncertainties. Monte Carlo analysis for parameter identification is a Bayesian approach that calculates posterior parameter distributions. These distributions can then be used to determine parameter correlations, identifiability, and they can be sampled to estimate prediction uncertainty. However, naive Monte Carlo Analysis scales poorly with increasing numbers of parameters (Gilks and Roberts, 2020). In the last few decades, advances in MCMC (Geyer, 1992) have allowed Bayesian approaches to parameter identification, and inverse problems in general, to become very efficient (Villaverde and Banga, 2014). Thus, more computationally expensive models and models with more free parameters are able to be used with MCMC. There has been extensive work on improving MCMC even further for models with large numbers of parameters. Hug et al. (2013) demonstrated an MCMC method that can be efficiently used to calculate prediction uncertainties from a model with hundreds of parameters. Vanlier et al. (2012) used a profile likelihood approach to remove structurally unidentifiable parameters, followed by an MCMC approach that can run efficiently on the reduced system. The profile likelihood method is an approach that should not be left out in the discussion of identifiability. Raue et al. (2009) developed a very effective profile likelihood approach for analysing parameter identifiability that can be more efficient than MCMC in certain applications (Simpson et al., 2020), and has been shown to be more effective than popular Fisher information based approaches (Wieland et al., 2021). MCMC, however, allows the calculation of prediction uncertainties, and therefore is more suitable for the task-specific application detailed in our work. MCMC can also be easily adapted to new proposal steps, prior distributions, and various types of regularisation to make the calibration process more task specific (Gupta et al., 2020).

This paper describes a pipeline for automatic calibration of general cardiovascular system models to a patient-specific set of clinical data. This pipeline improves the efficiency of typical MCMC approaches, and therefore improves on the clinical usability of these techniques. Numerical experiments are conducted where we apply the pipeline to a specific lumped parameter cardiovascular system model with different sets of clinical data to obtain a clinically informative cerebral pressure prediction. The paper outline is as follows. Section 2.1 describes the parameter identification pipeline used for model calibration. Section 2.2 introduces the lumped parameter cardiovascular system model, the implementation details, and the clinical measurement data used for calibration. The calibration results for three patients are shown in Section 3, and we discuss the advantages, limitations, and future work in Section 4.

## 2 Materials and methods

### 2.1 Automated calibration pipeline

The parameter identification task is stated as finding the model parameters $\theta =\left[\theta_{1},\ldots ,\theta_{n_{\theta }}\right]$ such that the model predictions best match a set of target measurements $\hat{z}=\left[{\hat{z}}_{1}\ldots ,{\hat{z}}_{n_{z}}\right]$ in terms of the weighted sum of squared differences, i.e.,

$$\theta =\underset{\tilde{\theta }\in \Theta }{arg\;min}\left(\overset{n_{z}}{\underset{i=1}{\sum }}{\left(\frac{f_{i}\left(\tilde{\theta }\right)-{\hat{z}}_{i}}{\sigma_{i}}\right)}^{2}\right)$$ (1)

where $\Theta \subset R^{n_{\theta }}$ is the set of parameters in the physiological range, $\sigma =\left[\sigma_{1},\ldots ,\sigma_{n_{z}}\right]$ is the measurement standard deviation, and $f=\left[f_{1}\ldots ,f_{n_{z}}\right]$ is the proposed cardiovascular model that predicts estimates, ***z***, of the target measurements.

In order to determine the prediction uncertainties, we elect to use an MCMC approach. However, MCMC can require long run times to reach convergence. Therefore, to enhance efficiency of the MCMC analysis, the pipeline involves first conducting a sensitivity analysis of ***f*** with respect to ***θ*** to reduce the parameter set and to ensure local structural identifiability. This is followed by the use of MCMC to analyse the prediction uncertainty. This process of improving the run time of the MCMC analysis improves the clinical usability of the calibration process. The algorithm for this process is shown at the end of this section in Algorithm 1. The steps of the pipeline are:

1) Solve one instance of Eq. 1.
2) Determine the parameters structural identifiability with a sensitivity analysis.
3) Fix the parameters that are not structurally identifiable.
4) Repeat steps 1–3 until the model is structurally identifiable.
5) Run MCMC on the model with reduced dimension.
6) Sample the parameter posterior distributions and run the model to obtain prediction uncertainties.

#### 2.1.1 Sensitivity analysis/structural identifiability

This section details the sensitivity analysis approach that is used to reduce the parameter set by fixing structurally unidentifiable parameters (see Algorithm 1, lines 1–20). The sensitivity analysis method (Brun et al., 2001) is used to analyse the local sensitivities of the model to characterise the local structural identifiability.

First, an in-house genetic algorithm is used to find an approximate solution to Eq. 1. This gives the locally optimal parameter vector, ***θ*** *. Second, first-order finite differences are used to calculate a sensitivity matrix, $S\in R^{n_{z}\times n_{\theta }}$ , as

$$S_{kl}=\frac{\partial f_{k}\left(\theta^{*}\right)}{\partial \theta_{l}}\frac{\theta_{l}^{*}}{\sigma_{k}}\;\left(\text{no implied summation}\right),$$ (2)

where *f*_*k*_ are the model outputs. The second term in Eq. 2 normalises the sensitivity matrix with respect to the best fit parameter values and the measurement standard deviation. The parameter importance of each parameter, *δ*_*l*_, which is a measure of parameter *l*’s influence on all of the outputs, is then calculated with

$$\delta_{l}=\sqrt{\frac{1}{n_{z}}\overset{n_{z}}{\underset{k=1}{\sum }}S_{kl}^{2}}.$$ (3)

To analyse the collinearity of the parameters, first, each column of ***S*** is normalised by its corresponding parameter importance to give

$${\tilde{S}}_{kl}=\frac{S_{kl}}{\delta_{l}\sqrt{n_{z}}}\;\left(\text{no implied summation}\right).$$ (4)

Then, ${\hat{S}}^{ij}\in R^{n_{z}\times 2}$ is defined as the matrix with two columns equal to the *i*’th and *j*’th columns of $\tilde{S}$ . Following this, $N^{ij}\in R^{2\times 2}$ can be calculated,

$$N^{ij}={\left({\hat{S}}^{ij}\right)}^{T}{\hat{S}}^{ij}.$$ (5)

The smallest eigenvalue, (*μ*_*ij*_), of ***N*** ^*ij*^ is then calculated, which allows the calculation of the collinearity metric between parameters *i* and *j*, as

$$\gamma_{ij}=\frac{1}{\sqrt{\mu_{ij}}}.$$ (6)

This collinearity metric is simply called the collinearity throughout this paper. The collinearity for each parameter is compared to the collinearity threshold (see Algorithm 1, line 10) to determine structural unidentifiability. Also, the collinearity and parameter importance are determined for the parameters with respect to the core predictions, as well as the observable outputs. These values are compared against the parameter importance and collinearity thresholds to check if the parameters can be safely fixed without significantly reducing the prediction uncertainties. For pairs of parameters that have collinearity greater than the threshold, the parameter with the lowest importance is removed from the set of parameters to be identified and it is held fixed at an approximate physiological value. In addition, if there is a parameter with importance below the parameter importance threshold, that parameter is also fixed. The process of fitting parameters then removing the ones that are structurally unidentifiable is repeated until all parameters satisfy the chosen thresholds for collinearity and parameter importance. The parameter importance and collinearity thresholds are user defined values. However, the values of *t*_*i*_ = 0.1, and *t*_*c*_ = 10 used in this work seem to be effective for multiple applications. The threshold on prediction uncertainty will be clinician and task dependent. Therefore, determining whether the uncertainty is acceptable is not included in this pipeline.

#### 2.1.2 Quantifying uncertainties in model predictions

After removing the structural unidentifiabilities in the model, MCMC is used to determine parameter posterior distributions. The posterior distributions are then sampled and the forward simulation is solved to give the distribution and uncertainties of the core predictions. These uncertainties are available to be analysed by a researcher or clinician to give a task-specific assurance of acceptable model calibration. To take advantage of well-validated, parallel MCMC software, we chose to use emcee (Foreman-Mackey et al., 2013), a software for MCMC that is designed to run efficiently with minimal user interaction. The ensemble sampler of emcee was used for the analysis, with 32 parallel chains of 5,000 steps. A burn-in of 2,500 steps was used to discard steps dependant on the initial parameter values. The MCMC chain convergence was confirmed with the Geweke test (Geweke, 1992), where the *p*-values from the *Z*-test were greater than 0.05. Running the MCMC algorithm with the full parameter set for 100 h resulted in the Geweke test failing, demonstrating that using MCMC without parameter set reduction is less tractable than the proposed approach.

The OpenCOR software (Garny and Hunter, 2015) which uses the CVODES solver (Serban and Hindmash, 2005), was used for solving the model system of ODE’s. If CVODES failed to run during a simulation, the parameter set was rejected in both the genetic algorithm and MCMC.

Algorithm 1 Automatic Calibration Algorithm. Notation: genetic_algorithm_Θ_ searches an estimate of parameters ***θ*** ∈ Θ; *ϵ*_*i*_ is the *i*th vector of the canonic basis; Θ/spanQ is the quotient space of Θ by spanQ , i.e., it is the reduced space after fixing the parameters related to the canonic vectors in Q ; *δ*_*i*_ is the parameter importance related to *θ*_*i*_; *γ*_*ij*_ is the collinearity between parameters *θ*_*i*_ and *θ*_*j*_; $\delta_{i}^{p}$ is the parameter importance with respect to the core predictions; $\gamma_{ij}^{p}$ is the collinearity of the parameters with respect to the core predictions;*ϕ* is the empty set; *t*_*i*_, *t*_*c*_, and *t*_*u*_ are the thresholds related to the parameter importance, parameter collinearity, and prediction uncertainty, respectively; ***θ***_*dist*_ is the vector of parameter posterior distributions; $\theta_{\mathrm{dist}}^{\mathrm{sample}}$ is a matrix of parameter vectors sampled from the posterior distribution; ***θ***_*init*_ is a matrix of parameter vectors for initialising the MCMC chains; N is the normal distribution; *σ*_*init*_ is the standard deviation of the random noise applied to initialise ***θ***_*init*_ for MCMC (*σ*_*init*_ = 0.01 ***θ*** in this paper); *f*_*pred*_ are the output predictions of the model.

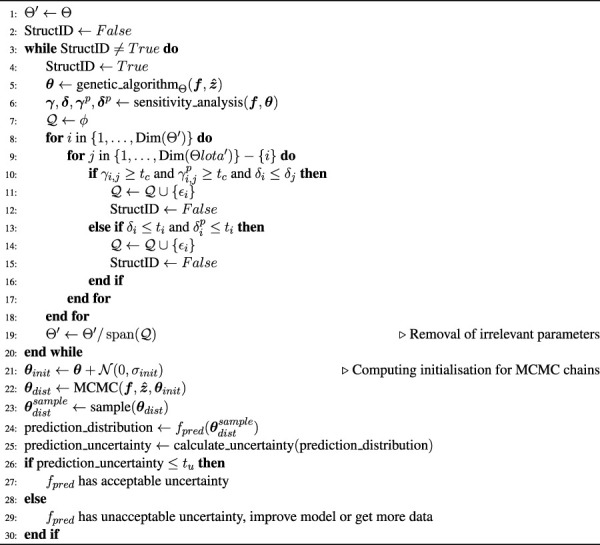

### 2.2 Application to a cardiovascular system model

This section briefly describes the CVS model that we calibrate in the numerical experiments of our pipeline. It also details the patient data that we use for calibration.

#### 2.2.1 Cardiovascular system model

**FIGURE 1:**
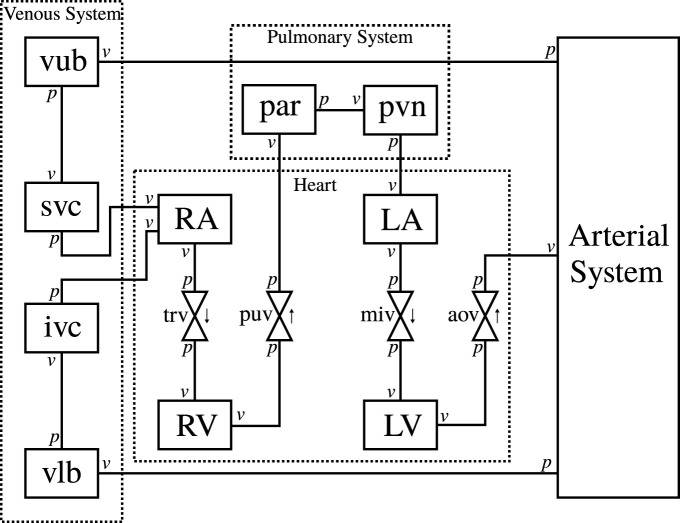
Schematic of the circulatory system model. RA, right atrium, RV, right ventricle, LA, left atrium, LV, left ventricle, trv, tricuspid valve, puv, pulmonary valve, miv, mitral valve, aov, aortic valve, par: pulmonary artery, pvn: pulmonary vein, vub, venous upper body, svc, superior vena cava, vlb, venous lower body, ivc, inferior vena cava, *v*, flow boundary condition, *p* pressure boundary condition.

Figure 1 shows the modular CVS model we have designed to be applicable for modelling different cardiovascular events in health and disease. Lumped parameter BG models are selected here for their ease of use, computational efficiency, and ability to be modularised to create arbitrary topology circulatory system models for specific clinical applications.

The arterial system is designed to be modular and adaptable. An in-house software (Argus and Maso Talou, 2022) generates a combined model of modular BG sections from the definition of a network of arterial vessels. The arterial network used in this paper is shown in Appendix Figure A1 (Supplementary Data Sheet 2). The modules for vessels, heart chambers, and valves are also described in the Appendix.

#### 2.2.2 Pipeline application

For the parameter reduction step of the pipeline, the parameter importance was set to a low value of *t*_*i*_ = 0.1 to ensure only the very low importance parameters would be removed. The collinearity threshold was set to *t*_*c*_ = 10, which agreed with Brun et al. (2001), who found that the critical threshold for collinearity is between 5 and 20. For this application, a core prediction of the pressure in the middle cerebral artery was chosen as the location for cerebral pressure prediction. Once the parameter posterior distributions were determined, 100 parameter values were randomly sampled from the final parameter distribution, and the model was simulated for each sample to give a distribution of the core pressure prediction.

In the software developed for this paper, the prior distributions have a default setting to be uniform between pre-defined, non-negative physiological ranges. However, they are modifiable to allow more accurate priors if more information is known. The pre-defined physiological parameter ranges used in this paper are available from https://github.com/FinbarArgus/circulatory_autogen/blob/automatic_parameter_id_paper_release/resources/physiological_params_for_id.csv and the units, physiological values for all fixed parameters, and the references for parameter values are available from https://github.com/FinbarArgus/circulatory_autogen/blob/automatic_parameter_id_paper_release/resources/physiological_parameters.csv.

#### 2.2.3 Clinical data

To demonstrate the application of the framework to experimental data, echocardiographic measurements for three patients with normal systolic function were used for calibration of the CVS model (see Table 1). Minimum and maximum atrial and left ventricular volumes over one cardiac cycle were calculated by tracing the endocardial border on apical echocardiographic images, according to standard clinical guidelines (Lang et al., 2015). Non-invasive systolic and diastolic pressures, as well as an estimate of mean arterial pressure derived from suprasystolic pressure waveforms (Lin et al., 2012) were obtained with a sphygmomanometer (BP+, Uscom, Sydney, Australia) on the upper arm. Ethical approval for this study was granted by the Health and Disability Ethics Committee of New Zealand (reference 17/NTB/46), and written, informed consent was obtained from each participant.

**TABLE 1:** Baseline patient characteristics including age, sex (M = male, F = female), height, weight, and resting systolic (sys.) and diastolic (dia.) blood pressure (BP).

Id	Age (years)	Sex	Height (m)	Weight (kg)	BP (sys./dia. MmHg [kPa])
Patient 1	45	M	1.70	79	112/73 [14.9/9.9]
Patient 2	34	F	1.67	89	137/80 [18.3/10.7]
Patient 3	49	F	1.67	100	114/77 [15.2/10.3]

To be able to estimate accurate uncertainties of our core predictions, the uncertainties of our measurements must be taken into account. For this, we use the coefficient of variation (CV) for repeated inter-observer measurements from the literature. We use inter-observer variability from each reference to maximise the sources of error that we take into account. Table 2 details the CV values that are used to calculate a standard deviation for each patient. The CV is related to the measurement standard error (*σ*) as

$$CV=\frac{RC\%}{2.77}=\frac{\sigma }{\hat{z}}$$ (7)

where RC% is the repeatability coefficient as a percent of the mean/measurement value and $\hat{z}$ is the measurement value.

**TABLE 2:** Coefficient of variation (CV) for each measurement, with data sources. *^1^: taken from 3D RC%, *^2^: variability between measurements separated by 24 h, *^3^: estimated as average of $\max\left(p_{BR_{L}}\right)$ and $\min\left(p_{BR_{L}}\right)$ , *^4^: assumed to be the same as for MC, *^5^: non-crucial flow rates are assumed to have a high CV.

Measurement	Description	CV	Source
max (*q*_*la*_)	max left atrium volume	21.3%	Letnes et al. (2021)
min (*q*_*la*_)	min left atrium volume	23.5%*^1^	Letnes et al. (2021)
max (*q*_*lv*_)	max left ventricle volume	12.6%	Zhao et al. (2021)
min (*q*_*lv*_)	min left ventricle volume	19.4%	Zhao et al. (2021)
max (*q*_*ra*_)	max right atrium volume	10.0%	Van Der Zwaan et al. (2011)
$\max\left(p_{BR_{L}}\right)$	max brachial pressure	1.5%*^2^	Abellán-Huerta et al. (2018)
$\min\left(p_{BR_{L}}\right)$	min brachial pressure	4.0%*^2^	Abellán-Huerta et al. (2018)
$mean\left(p_{BR_{L}}\right)$	mean brachial pressure	2.25%*^3^	Abellán-Huerta et al. (2018)
$mean\left(v_{LE_{RT}}\right)$	mean leg terminal flow	30.0%*^5^	Assumed large
$mean\left(v_{BR_{RT}}\right)$	mean brachial terminal flow	30.0%*^5^	Assumed large
$mean\left(v_{PC_{RT}}\right)$	mean posterior cerebral terminal flow	8.0%*^4^	Wen et al. (2019)
$mean\left(v_{EC_{RT}}\right)$	mean external carotid terminal flow	8.0%*^4^	Wen et al. (2019)
$mean\left(v_{MC_{RT}}\right)$	mean middle cerebral terminal flow	8.0%	Wen et al. (2019)
$mean\left(v_{AC_{RT}}\right)$	mean anterior cerebral terminal flow	8.0%*^4^	Wen et al. (2019)
$mean\left(v_{TR_{T}}\right)$	mean trunk flow	50.0%*^5^	Assumed large

To approximate the cerebral blood flow that will be acquired in the future, mean flow values at the terminals of our model were obtained from the ADAN-218 model (Blanco et al., 2015). The flows at the arm, leg, and trunk terminals are included as synthetic measurements from ADAN to give the fitting process an approximate target flow for each region. Matching a patients flow in these inferior regions is assumed to be less important for predicting cerebral haemodynamics. Therefore, their CV are set to large values to reduce the influence of their synthetic flow measurements on the fitting process.

**FIGURE 2:**
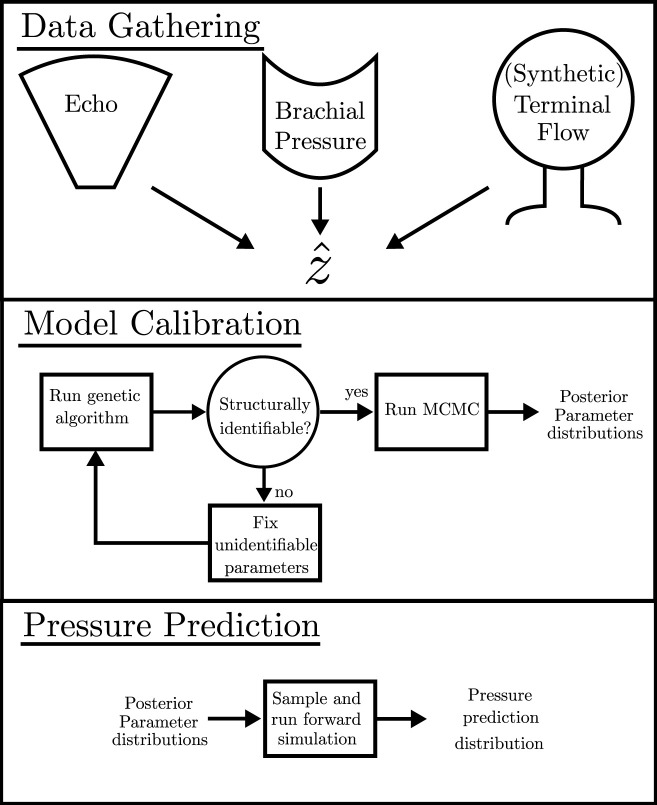
Schematic detailing the pipeline for required data, the calibration process, and the calculation of the pressure prediction distribution.

The measurements in Table 2 are the $\hat{z}$ measurements used as the ground truth for fitting the model. Each measurement also has a *σ*_*i*_ calculated from the CV in Table 2 that influences the minimisation in Eq. 1 for both the genetic algorithm and the MCMC analysis. The term inside the outer bracket of Eq. 1 is the cost function for the genetic algorithm, and the likelihood for MCMC is −0.5 times the cost function. A simple schematic of the full pipeline is shown in Figure 2. The physiological parameter ranges mentioned in Section 2.2.2 are used as parameter limits for the genetic algorithm and as the limits on the uniform prior for the MCMC analysis.

## 3 Results

**FIGURE 3:**
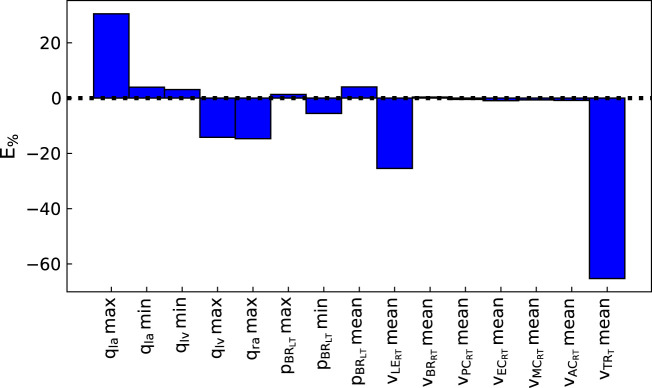
Percentage error between optimal model outputs (*f*_*i*_(***θ***)) and measured data $\left({\hat{z}}_{i}\right)$ from the parameter identification pipeline for Patient 1 (normalised by ${\hat{z}}_{i}$ ). *q*_*la*_: left atrium volume, *q*_*lv*_: left ventricle volume, *q*_*ra*_: right atrium volume, $p_{BR_{L}}$ : left brachial pressure, $v_{LE_{RT}}$ : right leg terminal flow, $v_{BR_{RT}}$ : right brachial terminal flow, $v_{PC_{RT}}$ : right posterior cerebral terminal flow, $v_{EC_{RT}}$ : right external carotid terminal flow, $v_{MC_{RT}}$ : right middle cerebral terminal flow, $v_{AC_{RT}}$ : right anterior cerebral terminal flow, $v_{TR_{T}}$ : trunk terminal flow.

**FIGURE 4:**
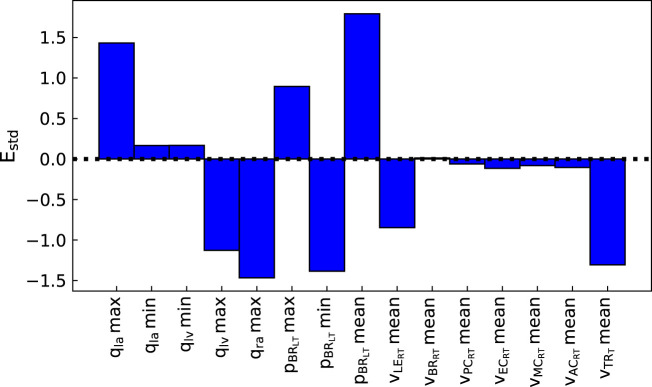
Normalised error between optimal model outputs (*f*_*i*_(***θ***)) and measured data $\left({\hat{z}}_{i}\right)$ from the parameter identification pipeline for Patient 1 (normalised by *σ*_*i*_). *q*_*la*_: left atrium volume, *q*_*lv*_: left ventricle volume, *q*_*ra*_: right atrium volume, $p_{BR_{L}}$ : left brachial pressure, $v_{LE_{RT}}$ : right leg terminal flow, $v_{BR_{RT}}$ : right brachial terminal flow, $v_{PC_{RT}}$ : right posterior cerebral terminal flow, $v_{EC_{RT}}$ : right external carotid terminal flow, $v_{MC_{RT}}$ : right middle cerebral terminal flow, $v_{AC_{RT}}$ : right anterior cerebral terminal flow, $v_{TR_{T}}$ : trunk terminal flow.

The pipeline described in Algorithm 1 was applied to measurements from three patients, described in Section 2.2.3, to analyse the suitability of the proposed automated approach for obtaining a cerebral arterial pressure prediction with its corresponding uncertainty. Figures 3, 4 show the percentage error and the error normalised by the measurement standard deviation for optimal model outputs compared with the measurements. The approach provided fitting error for each output within ±2 ***σ*** for Patients 1 and 3, and within ±3 ***σ*** for all but one output of Patient 2. Figures 3, 4 show results for Patient 1, and the figures in the Supplementary Material show results for Patients 2 and 3. The large error in the flow in the trunk terminal (*v*_*TR*_) (see Figure 3) shows that the error between model output and measurement is heavily dependent on the measurement standard deviation. The plots for each of the three patients and for all of the outputs are provided in the Supplementary Material. The fitting errors for each patient are reasonably consistent, thus showing robust applicability to the different data sets (see Table 3).

**TABLE 3:** Parameter estimates and standard deviations, error of the model output versus the measured data, and the core prediction values with confidence intervals. The (fixed) parameters were set to physiological values as they were not structurally identifiable. N/A indicates measurements that were not obtained for that patient.

	Patient 1	Patient 2	Patient 3
Parameters			
*q*_*sbv*_ [*ml*]	1041 ± 45	1204 ± 59	1013 ± 55
*E*_*LVa*_ [*MPa*/*m* ^3^]	341 ± 59	219 ± 46	308 ± 105
*E*_*RVa*_ [*MPa*/*m* ^3^]	73.3 (fixed)	73.3 (fixed)	73.3 (fixed)
*R*_*par*_ [*MPa*.*s*/*m* ^3^]	10.7 (fixed)	10.7 (fixed)	10.7 (fixed)
$R_{LE_{T}}$ [*MPa*.*s*/*m* ^3^]	2078 ± 498	2,227 ± 656	1090 (fixed)
$R_{BR_{T}}$ [*MPa*.*s*/*m* ^3^]	7178 ± 1405	8709 ± 976	7349 ± 1413
$R_{AC_{T}}$ [*MPa*.*s*/*m* ^3^]	6954 ± 627	8289 ± 657	7234 ± 619
$R_{EC_{T}}$ [*MPa*.*s*/*m* ^3^]	4164 ± 363	4960 ± 411	4362 ± 382
$R_{MC_{T}}$ [*MPa*.*s*/*m* ^3^]	4452 ± 396	5,307 ± 467	4650 ± 370
$R_{PC_{T}}$ [*MPa*.*s*/*m* ^3^]	19192 ± 1698	23146 ± 1956	20100 ± 1778
$R_{TR_{T}}$ [*MPa*.*s*/*m* ^3^]	880 (fixed)	938 ± 334	1335 ± 319
*C*_*V*_ [*m* ^3^/*MPa*]	0.5 (fixed)	0.5 (fixed)	0.5 (fixed)
Output Error			
max (*q*_*la*_)	30.5%	55.4%	29.8%
min (*q*_*la*_)	3.93%	N/A	14.7%
max (*q*_*lv*_)	14.2%	21.1%	N/A
min (*q*_*lv*_)	3.1%	7.8%	N/A
max (*q*_*ra*_)	14.7%	21.9%	17.4%
$\max\left(p_{BR_{R}}\right)$	1.34%	0.23%	2.6%
$\min\left(p_{BR_{R}}\right)$	5.54%	9.11%	3.7%
$mean\left(p_{BR_{R}}\right)$	4.03%	11.1%	4.6%
$mean\left(v_{LE_{RT}}\right)$	25.4%	14.1%	13.9%
$mean\left(v_{BR_{RT}}\right)$	0.38%	2.4%	3.6%
$mean\left(v_{PC_{RT}}\right)$	0.49%	2.8%	0.6%
$mean\left(v_{EC_{RT}}\right)$	0.93%	2.7%	0.02%
$mean\left(v_{MC_{RT}}\right)$	0.65%	3.6%	0.7%
$mean\left(v_{AC_{RT}}\right)$	0.84%	3.7%	1.0%
$mean\left(v_{TR_{T}}\right)$	65.3%	60.0%	75.9%
Prediction			
$\max\left(p_{MC_{R}}\right)$ [*kPa*]	$14.5_{13.7}^{15.3}$	$16.7_{16.1}^{17.4}$	$14.3_{13.0}^{15.5}$
$\min\left(p_{MC_{R}}\right)$ [*kPa*]	$8.8_{8.4}^{9.3}$	$10.8_{10.3}^{11.4}$	$9.4_{9.0}^{9.9}$
$mean\left(p_{MC_{R}}\right)$ [*kPa*]	$11.1_{10.6}^{11.5}$	$13.2_{12.7}^{13.7}$	$11.5_{11.1}^{12.0}$

**FIGURE 5:**
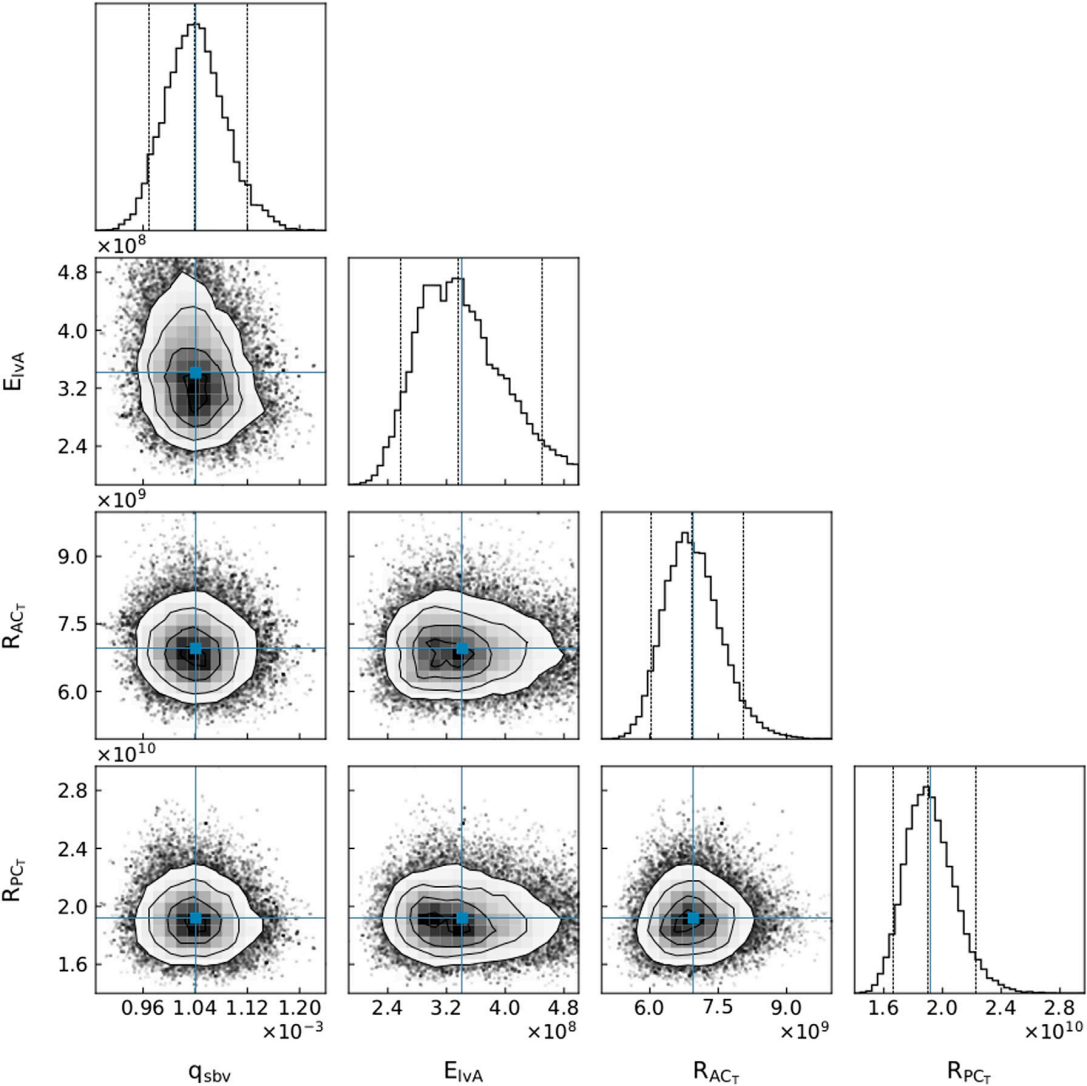
A subset of parameter distributions for Patient 1. *q*_*sbv*_: stressed blood volume, *E*_*lvA*_: left ventricle maximum elastance, $R_{AC_{T}}$ : anterior cerebral terminal resistance, $R_{PC_{T}}$ : posterior cerebral terminal resistance.

**FIGURE 6:**
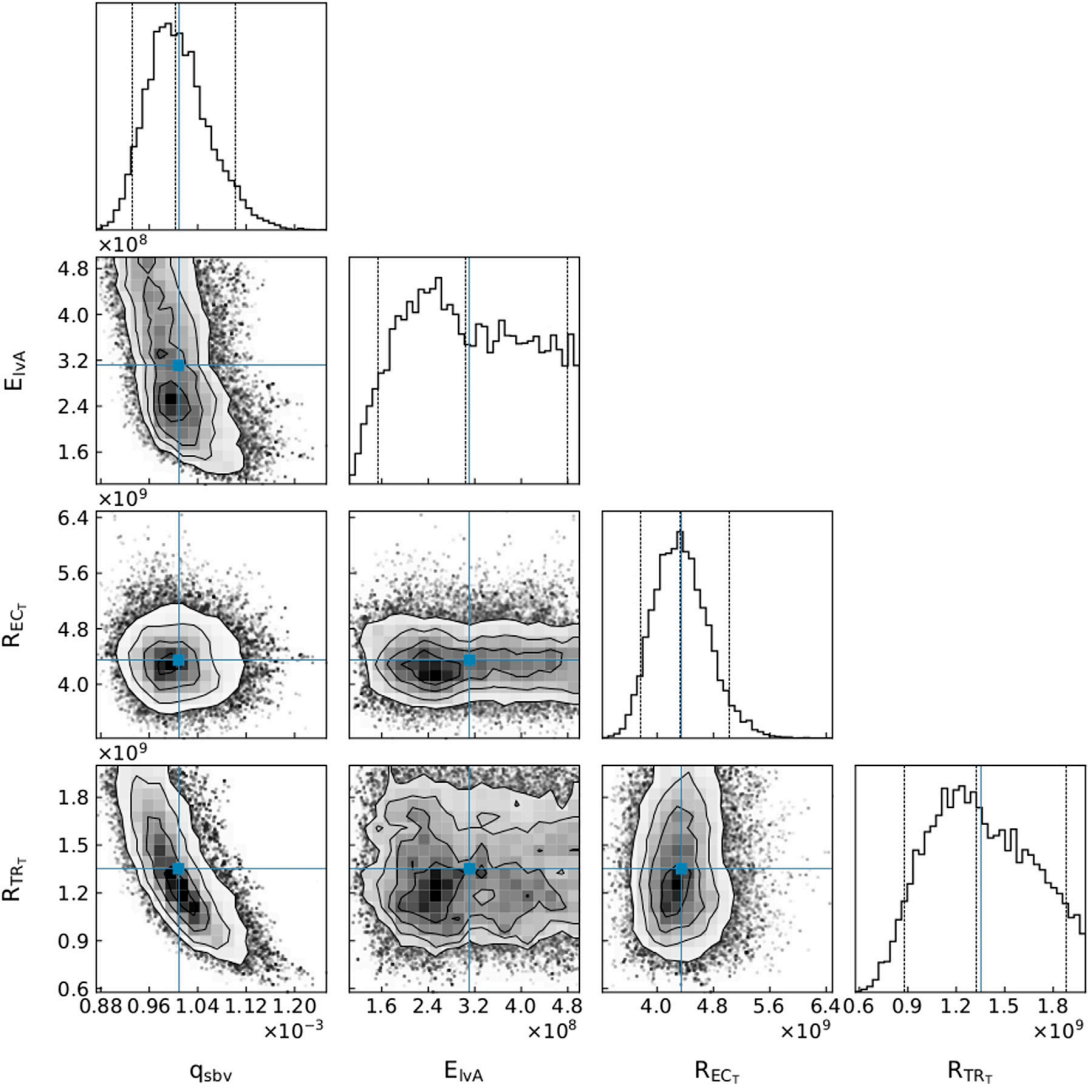
A subset of parameter distributions for Patient 3. *q*_*sbv*_: stressed blood volume, *E*_*lvA*_: left ventricle maximum elastance, $R_{EC_{T}}$ : external carotid terminal resistance, $R_{TR_{T}}$ : trunk terminal resistance.

The parameter distributions from the MCMC method revealed that all parameters were clearly practically identifiable for Patient 1 (see Figure 5), whereas for Patient 3 *E*_*lvA*_ was practically unidentifiable for the chosen parameter range (see Figure 6).

**FIGURE 7:**
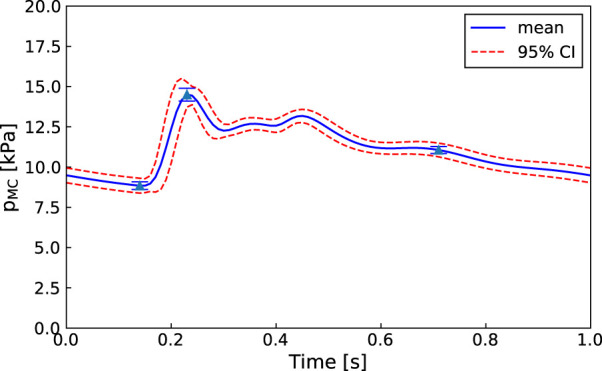
Patient 1: Mean, standard deviation, and 95% confidence interval for the right middle cerebral artery pressure predictions $\left(p_{MC_{R}}\right)$ sampled from the MCMC posterior parameter distributions (100 samples).

**FIGURE 8:**
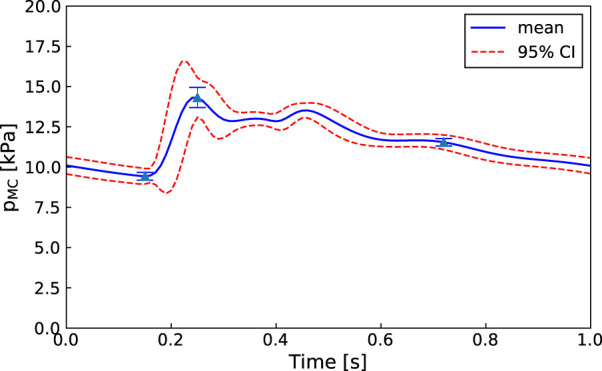
Patient 3: Mean, standard deviation, and 95% confidence interval for the middle cerebral artery pressure predictions $\left(p_{MC_{R}}\right)$ sampled from the MCMC posterior parameter distributions (100 samples).

An advantage of our approach is that, as in Figure 6, even if one of the parameters is not practically identifiable (i.e., there is a flat region in its posterior distribution), the calibrated model may still be sufficient for estimating the core predictions (see Figures 7, 8). Our analyses show that prediction uncertainties attributed to parameter estimation can be automatically generated for this CVS model. If the uncertainty for Patient 3’s core prediction is acceptable to the clinician, then this approach provides a task-specific acceptable model calibration, even though it has practically unidentifiable parameters. In Section 4, we discuss the need to improve this uncertainty estimation to account for the error between the model and the data, therefore providing a better representation of the uncertainty of the prediction and making the uncertainty more interpretable for a clinician.

## 4 Discussion

The proposed methodology is focused on improving the clinical viability of model calibration approaches for predicting non-observed quantities of the CVS. An approach that can be used clinically must be efficient, robust, and require minimal user input. We aim to improve the efficiency of MCMC calibration processes by using methods such as parallel genetic algorithms and emcee that are computationally efficient for HPC use and by reducing the parameter set to make it structurally identifiable before running MCMC. Additionally, both the in-house genetic algorithm and emcee (Foreman-Mackey et al., 2013) are designed to minimise required user input. The robustness of the approach is demonstrated by showing that each of the patients have middle cerebral artery peak pressure prediction uncertainties within ±10 mmHg (±1.3 kPa) (see Figures 7, 8 for Patients 1 and 3).

We have demonstrated the application of this approach to three patients with differing sets of available data (see Table 3). A set of structurally identifiable parameters was obtained for each set of data, demonstrating the robustness of the approach to varying availability of data. This is crucial for clinical application, as patients often have differing sets of measured data, due to availability of equipment, time constraints, and the inability to take certain measurements for certain patients.

### 4.1 Model reduction

The choice of setting parameters to fixed values to enhance structural identifiability could fictitiously decrease the estimated prediction uncertainty. In the current work, we assumed that this decrease in uncertainty was negligible. In future, rather than simply fixing parameters, more advanced model reduction steps could be applied to ensure that the model being used has a minimal number of parameters that are more identifiable. Any step made to reduce model complexity, in order to increase structural identifiability, has the risk of decreasing model accuracy as a result of the simplified dynamics. The reduction of free parameters is a compromise between providing sufficient model fidelity, and having parameters that are structurally identifiable. We argue that an approach such as ours that simply removes functional relations between model parameters by fixing some of the parameters may be all that is necessary for calculating realistic prediction uncertainties in many situations.

When a model has collinearities, fixing a parameter does not locally reduce the fidelity of the model. Instead, it only collapses a collinear relationship, so the free parameter affects the outputs in a way that both parameters could affect the outputs previously. It should be noted that this may reduce the fidelity of the model, due to no longer incorporating potential nonlinear global dynamics of the fixed parameter. However, if we assume that the local collinear behaviour approximately represents the global behaviour, fixing all structurally unidentifiable parameter does not reduce model fidelity for fitting to data. However, reducing the parameter set further has the risk of significantly reducing model fidelity and causing the prediction uncertainties to be fictitiously reduced. Therefore, we have chosen not to reduce the parameter set once it is structurally identifiable, meaning that we do not use the prediction uncertainty to further reduce the model.

The assumption that the local structural identifiability represents the global structural identifiability is a shortcoming of this model that can be improved in future work, such as by implementing a global structural identifiability method (Dobre et al., 2010; Joubert et al., 2020).

The approach in this study is designed to be efficient for large models with many parameters. MCMC can be burdensome when used with a large number of parameters, and this is particularly inefficient when the parameters have collinearities. The parameter set reduction obtained from the sensitivity analysis reduces the MCMC expense, resulting in a more tractable problem. Running without parameter reduction takes longer than 100 h, as demonstrated by the model with a full parameter set failing the Geweke test after 100 h of MCMC simulation. Therefore, the approach detailed in this paper is more clinically viable, in terms of efficiency, than naively running MCMC on the full parameter set. The profile likelihood approach (Raue et al., 2009) could instead be used to show practical identifiability, as other works in the literature have reported greater efficiency compared to MCMC (Simpson et al., 2020). However, the speed-up would be diminished or reversed when using slower non-gradient based optimisation methods, such as the genetic algorithm created in this work, because the profile likelihood approach requires many optimisation runs. The OpenCOR software (Garny and Hunter, 2015) used here uses the CVODES solver (Serban and Hindmash, 2005), which has the ability to calculate sensitivities with automatic differentiation (although this is not currently available through OpenCOR), therefore, faster gradient based optimisation methods could be used in the future. Access to the gradients would also improve the efficiency of the structural identifiability analysis, as the calculation of sensitivities using finite differences would not be necessary. This may also allow efficient time-dependent sensitivity analysis, as presented in Joubert et al. (2020).

Multiple groups have worked on reducing circulatory system models in a way that retains accurate prediction of the pressure wave. Epstein et al. (2015) showed that a 55 compartment arterial system can be reduced to 21 compartments with less than 2% average relative error. By additionally incorporating parameter identification in their reduction framework, Fossan et al. (2018) showed that to capture important features of the aortic waveform in a 1D blood flow model of the circulatory system, only minimal descriptions of the limbs and cerebral circulation were required. The present study uses a model with fixed topology. However, while conducting the current study we have developed a model generation software (Argus and Maso Talou, 2022) so that we now have a framework to begin to incorporate similar model reduction steps as (Epstein et al., 2015; Fossan et al., 2018) to automatically reduce the topology of the model.

### 4.2 Core predictions

An important part of our approach is the idea that the use of core predictions (Cedersund, 2012) enhances the task specificity of the approach in comparison to typical parameter identification approaches. A demonstration of this is presented in the cornerplots (Foreman-Mackey, 2016) in Figure 6, where $E_{lv_{A}}$ has a reasonably flat region in parameter space, indicating that it may be practically unidentifiable. This unidentifiability is likely due to the unavailability of *q*_*lv*_ measurements for Patient 3. Even with this supposed practical unidentifiability, our method provides pressure predictions with small uncertainty, as seen in Figure 8. Although the unidentifiability of $E_{lv_{A}}$ causes a slightly larger uncertainty during systole, if the uncertainty is within clinically acceptable limits, then we claim that the model is acceptably calibrated. Therefore, a model can be clinically useful even though it may have practically unidentifiable parameters. A core prediction with a user defined uncertainty is a task-specific metric that is typically less stringent than identifiability approaches that look at flatness of the parameter posterior distributions. Core predictions focus on what is of interest for application, i.e. the uncertainty of the desired prediction. Of course, the actual prediction uncertainty should also account for model approximation errors due to the model equations only being an approximation of the physiology.

The uncertainty estimate of the core prediction has been assumed to be unaffected by fixing structurally unidentifiable parameters. Cedersund and Roll (2009) stated that if the parameter space is reduced by fixing parameters, a reliable core prediction can no longer be obtained. We argue that unless you calculate and propagate the uncertainty of every parameter in the model, which is intractable for all but the simplest of models, any realistic approach to calculating a core prediction can only provide an approximation. In our approach, by ensuring that the fixed parameters do not significantly affect the core prediction, we can approximate its uncertainty due to parameter uncertainties.

Determining the uncertainties of core predictions provides users with valuable knowledge about how confidently they should treat the predictions. This fully automated approach is designed so that, if used clinically, it can indicate a degree of confidence on a core prediction with minimal input from the clinician. On the other hand, the MCMC distributions can inform a researcher about the parameters that may be less sensitive (i.e., present plateaus in the parameter posterior distributions) with respect to the cost function, and therefore provide information about practical unidentifiability. Consequently, even though this pipeline does not use practical identifiablity analysis to verify that the model calibration can be trusted, the parameter posterior distributions are available for visualisation by an interested party (see Figures 5, 6). These distributions can indicate whether parameters are unidentifiable, suggest whether the model could be reduced in a certain manner, or inform where more data is required to reduce parameter uncertainties.

### 4.3 Limitations and future work

Paun et al. (2020) describes an approach to use Gaussian processes to account for the uncertainty due to a mismatch between model outputs and measurements, which will be investigated when advancing the current approach. Due to not accounting for this so-called model mismatch, the uncertainty displayed in this study is a lower bound. The crucial information obtained is the size of the prediction uncertainty that is due to the parameter estimation process, i.e., it gives a task-specific alternative to practical identifiability approaches.

A significant limitation of the MCMC approach is that the choice of prior distributions can be important for accurate parameter estimation (Lambert et al., 2005), and thus for accurate predictions. We chose to assign uniform parameter distributions between manually-defined physiological parameter ranges that represent normal physiology. This approach has the advantage of being simple, and requires minimal user input. However, the use of uniform priors may hinder the convergence of the MCMC chains to their true posterior distributions. For the present relatively simple application, this was not a problem. However, in future clinical applications, parameter priors could be defined using sub-population data that more closely represents each specific patient to be analysed. For example, an older patient may have a higher prior distribution range for their terminal resistance compared to a younger patient, due to the known effect of arterial wall thickening with age. Such an age-dependent prior could be obtained from population studies.

Currently, this pipeline uses *in silico* data from the ADAN model to approximate mean flow measurements that can be obtained from 4D-flow MRI. This enabled the testing and refinement of the approach in the absence of suitable *in-vivo* data. One potential problem due to this use of synthetic data for the cerebral blood flow, is that it may not be compatible with the clinical echocardiographic measurements. However, as shown by our fitting to the data (see Table 3) and the successful Geweke convergence test, any potential discrepancies did not hinder convergence of the genetic algorithm or the MCMC approach. Additionally, we used ADAN flow values to approximate the flow in the arms, legs, and trunk, but in the future, flow measurements could be used for these locations. Identification of the terminal compliances, terminal moments of inertia, and venous resistances will be investigated in the future, when we fit the model to the dynamic brachial pressure and left ventricle volume curves.

In this work, the large chain lengths (5,000 steps × 32 walkers) required to reach convergence of the MCMC method resulted in calibration run-times of approximately 24 h on 16 Intel Xeon 3.0 GHz cores. Gaussian processes and surrogate models, as in Paun et al. (2020), can be used in future work to significantly reduce run-times to provide tractable in-clinic analyses.

The pipeline detailed in this work has been designed with the aim of creating a calibration process that can be used in conjunction with automated image processing tools, and our automatic model generation software. This will enable a pipeline from a magnetic resonance angiography (MRA) image, to a segmented and labelled vessel network, to a calibrated model of a patient’s haemodynamics, and finally to trusted circulatory pressure predictions.

## 5 Conclusion

This study details a task-specific approach to parameter identification that relaxes the constraints of having to have each parameter be practically identifiable. Instead, the pipeline calculates the prediction uncertainties, so that a researcher or clinician can decide whether they are within acceptable bounds. A sensitivity analysis is used to account for functional relationships between collinear parameters, in order to decrease the number of free parameters, and hence improve the efficiency of a subsequent MCMC analysis. The prediction uncertainty is then calculated by sampling from the parameter posterior distributions. This approach allows computationally efficient calibration of complex models to improve the clinical applicability of circulatory system modelling.

This approach has been applied to the calibration of a CVS model to clinically available cardiac ultrasound and synthetic cerebral blood flow data. We have shown that, for each patient, the model can be efficiently calibrated to give middle cerebral artery peak pressure prediction uncertainties within ±10 mmHg (±1.3 kPa).

Calibrating complex models to data is generally very difficult in the clinical setting. This approach provides advancements in the methods for clinical model calibration without compromising the accuracy for the intended clinical task.

## Data Availability

The original contributions presented in the study are included in the article/Supplementary Material, further inquiries can be directed to the corresponding author.

## Supplementary material

Data Sheet 1 Supplementary Tables and Figures

Data Sheet 2 Appendix
