# Supplementary material for: Automated model calibration with parallel MCMC: Applications for a cardiovascular system model
Source: Front Physiol. 2022 Nov 9;13:1018134. doi: 10.3389/fphys.2022.1018134 (PMC9683692; doi:10.3389/fphys.2022.1018134)
Supplement: Supplementary file 1 [file DataSheet2.pdf]

Appendix

This appendix details the lumped parameter CVS model used in this work, and the modular sections that can be used to generate an arbitrary topology circulatory system with our in-house software.

The modules in Figure A1 have flow (v) or pressure (p) boundary conditions. An important aspect of coupling BG modules is that where two modules are coupled, dual variables must be set as the boundary condition for each module. In the haemodynamic model, a module's pressure boundary must be connected to a flow boundary of the coupled module (see Figure A1), where each connection line between modules has one p-boundary condition and one v-boundary condition.

As introduced by Safaei et al. (2018), different modules have been proposed for each type of boundary condition. The BG diagrams for single input single output modules, *pv-type*, *pp-type*, and *vv-type* are presented in Figures A2 to A4.

The BG diagrams for the junction modules can be simply created by splitting the flow (v_b or v_{b2}) boundary condition into two flow boundary condition variables. This yields *v-vv-type*, *p-vv-type*, and *vv-vv-type* junction modules. As an example of splitting v_{b2} from Figure A4, Figure A5 shows the *v_vv-type* junction. The opposite input/output boundary conditions of these BG modules are not shown, as they are equivalent up to a choice of positive flow direction. The user choice of positive flow direction has not been shown in Figure A1, as it can be arbitrarily chosen. The model created with our model generation software, however, does define the user-chosen positive flow direction and is available at `https://github.com/FinbarArgus/circulatory_autogen/tree/automatic_parameter_id_paper_release`.

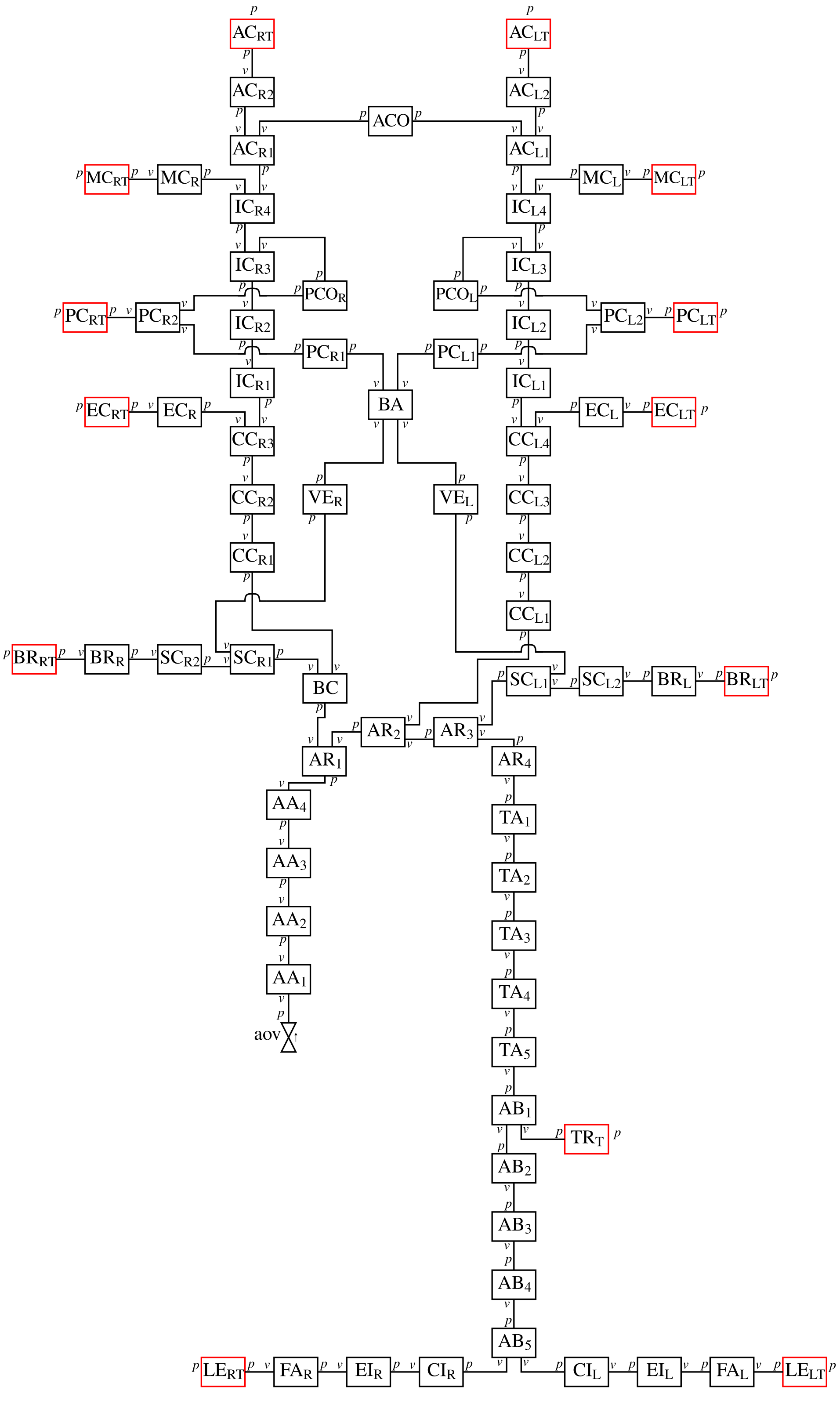

Figure A1. Schematic of the arterial system. AC: Anterior cerebral, MC: Middle cerebral, ACO: Anterior communicating, IC: Internal carotid, PCO: Posterior communicating , PC: Posterior cerebral, EC: External carotid, CC: Common carotid, BA: Basilar, VE: Vertebral, BR: Brachial, SC: Subclavian, BC: Brachiocephalic, AA: Ascending aorta, AR: Aortic arch, TA: Thoracic aorta, AB: Abdominal aorta, TR: Trunk terminal, CI: Common Iliac, EI: External iliac, FA: Femoral artery, LE: Leg terminal, $_{L/R}$: left/right, $_{T}$: terminal, p: pressure boundary condition, v: flow boundary condition

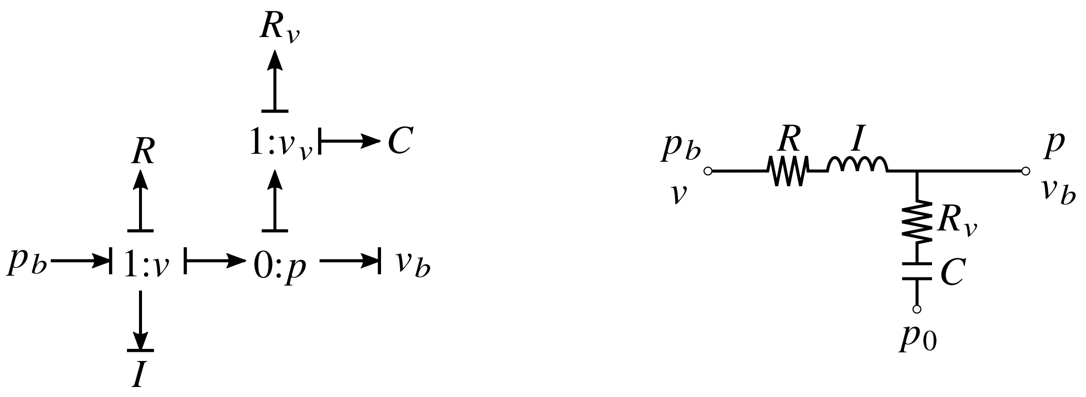

Figure A2. Bond graph and Windkessel diagram for the pressure-flow boundary condition (*pv-type*) module.

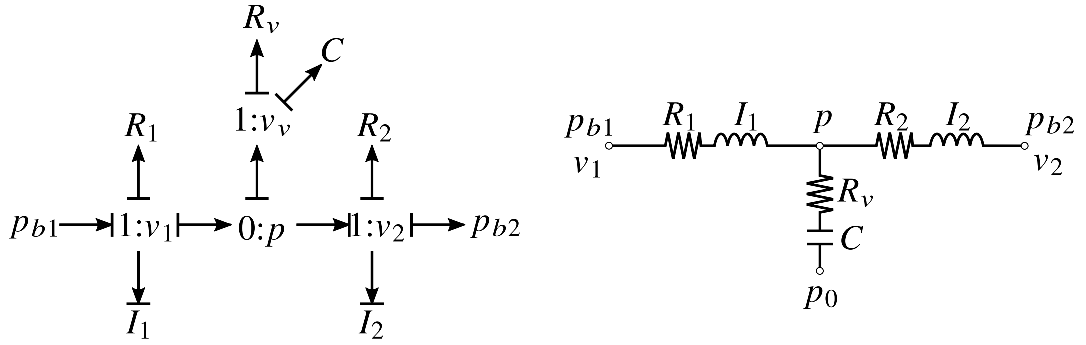

Figure A3. Bond graph and Windkessel diagram for the pressure-pressure boundary condition (*pp-type*) module.

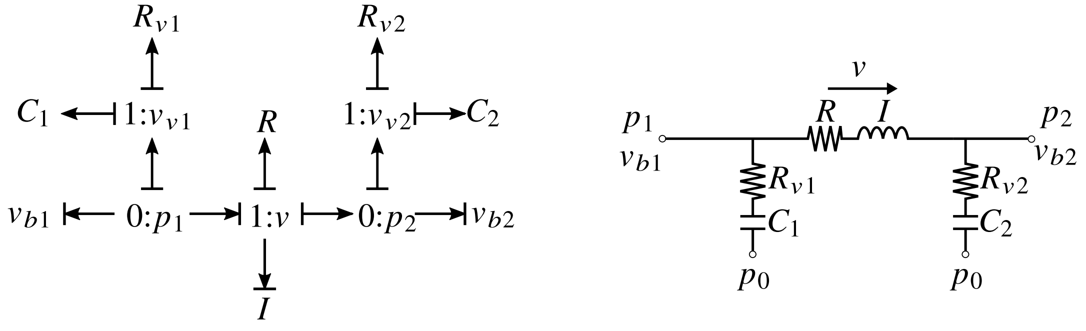

Figure A4. Bond graph and Windkessel diagram for the flow-flow boundary condition (*vv-type*) module.

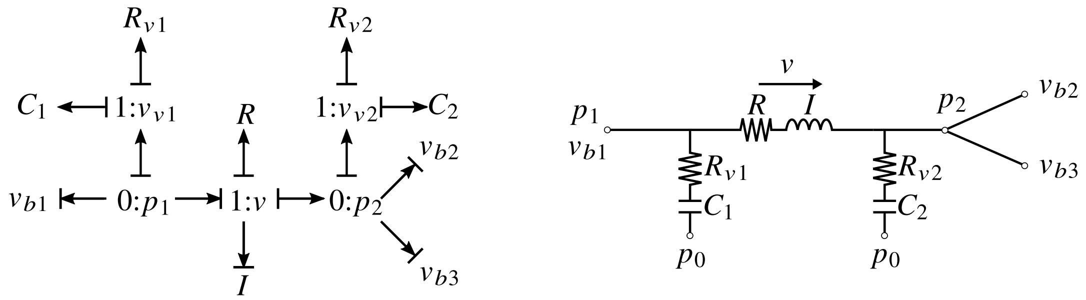

Figure A5. Bond graph and Windkessel diagram for the flow-flow boundary condition split junction module (*v-vv-type*).

The state space models for *pv-type*, *pp-type*, and *vv-type*, which were derived from the BG diagrams in Figures A2 to A4, are

$$\begin{aligned} \begin{bmatrix} \dot{q} \\ \dot{v} \end{bmatrix} &= \begin{bmatrix} 0 & 1 \\ -\frac{1}{CI} & -\frac{R}{I} \end{bmatrix} \begin{bmatrix} q \\ v \end{bmatrix} + \begin{bmatrix} -1 & 0 \\ 0 & \frac{1}{I} \end{bmatrix} \begin{bmatrix} v_b \\ p_b \end{bmatrix} \\ \begin{bmatrix} p \\ v \end{bmatrix} &= \begin{bmatrix} \frac{1}{C} & 0 \\ 0 & 1 \end{bmatrix} \begin{bmatrix} q \\ v \end{bmatrix} \end{aligned} \tag{S1}$$

$$\begin{aligned} \begin{bmatrix} \dot{q} \\ \dot{v_1} \\ \dot{v_2} \end{bmatrix} &= \begin{bmatrix} 0 & 1 & -1 \\ -\frac{1}{CI_1} & -\frac{R_1+R_{v1}}{I_1} & \frac{R_v}{I_1} \\ \frac{1}{CI_2} & \frac{R_{v1}}{I_2} & -\frac{R_2+R_{v2}}{I_2} \end{bmatrix} \begin{bmatrix} q \\ v_1 \\ v_2 \end{bmatrix} + \begin{bmatrix} 0 & 0 \\ \frac{1}{I_1} & 0 \\ 0 & -\frac{1}{I_2} \end{bmatrix} \begin{bmatrix} p_{b1} \\ p_{b2} \end{bmatrix} \\ \begin{bmatrix} v_1 \\ v_2 \end{bmatrix} &= \begin{bmatrix} 0 & 1 & 0 \\ 0 & 0 & 1 \end{bmatrix} \begin{bmatrix} q \\ v_1 \\ v_2 \end{bmatrix} \end{aligned} \tag{S2}$$

$$\begin{aligned} \begin{bmatrix} \dot{q_1} \\ \dot{q_2} \\ \dot{v} \end{bmatrix} &= \begin{bmatrix} 0 & 0 & -1 \\ 0 & 0 & 1 \\ \frac{1}{C_1 I} & -\frac{1}{C_2 I} & -\frac{R+R_{v1}+R_{v2}}{I} \end{bmatrix} \begin{bmatrix} q_1 \\ q_2 \\ v \end{bmatrix} + \begin{bmatrix} 1 & 0 \\ 0 & -1 \\ \frac{R_{v1}}{I} & \frac{R_{v2}}{I} \end{bmatrix} \begin{bmatrix} v_{b1} \\ v_{b2} \end{bmatrix} \\ \begin{bmatrix} p_1 \\ p_2 \end{bmatrix} &= \begin{bmatrix} \frac{1}{C_1} & 0 & 0 \\ 0 & \frac{1}{C_2} & 0 \end{bmatrix} \begin{bmatrix} q_1 \\ q_2 \\ v \end{bmatrix} \end{aligned} \tag{S3}$$

where q_i is the volume of the C_i compliance element.

Currently, in the terminal sections, the compliance (C) and moment of inertia (I) are set to small values to make the effects of terminal compliance and inertia insignificant. Similarly, the resistance of the vessels in the venous system are set to negligible values, assuming that the venous system has negligible resistance. See `https://github.com/FinbarArgus/circulatory_autogen/blob/automatic_parameter_id_paper_release/resources/ physiological_parameters.csv` for

the specific values set at each terminal and venous compartment. These small terms are left in the model, rather than removing them from the BG modules in Figures A2 to A4, to ensure the modules are general for when these effects need to be included.

In the non-terminal arteries, the vessels are assumed to be cylindrical and straight, and to consist of homogeneous, linear elastic material. For these assumptions, the compliance, resistance, and moment of inertia of each vessel segment is related to its radius by the following relations:

$$C = \frac{2\pi r^3 l}{hE}, \qquad R = \frac{8\mu l}{\pi r^4}, \qquad I = \frac{\rho l}{\pi r^2}, \tag{S4}$$

respectively, where r is the vessel radius, l is the vessel length, h is the wall thickness, E is the Young's modulus, and μ is the dynamic viscosity of the blood (Waite and Fine, 2007; Vlachopoulos et al., 2011). The wall thickness, h, is calculated from an empirical model,

$$h = r(ae^{br} + ce^{dr}), \tag{S5}$$

where $a = 0.2802$, $b = -505.3\,m^{-1}$, $c = 0.1324$, $d = -11.14\,m^{-1}$ were experimentally determined by Watanabe et al. (2013). The viscoelastic resistance related to the compliance, C, for each vessel is calculated with the following relation,

$$R_v = \frac{\tau_v}{C}, \tag{S6}$$

(see Westerhof and Noordergraaf (1970)) where τ_v is the time constant for stress relaxation, which is assumed to be $\tau_v = 0.01\,s$, following Safaei et al. (2018).

The heart model is a lumped parameter compliance model of the four chambers with four valves: namely, the tricuspid, pulmonary, mitral, and aortic valves. The state space model for each heart chamber is

$$\begin{aligned} \dot{q} &= \begin{bmatrix} 1 & -1 \end{bmatrix} \begin{bmatrix} v_{in} \\ v_{out} \end{bmatrix} \\ \begin{bmatrix} q \\ p \end{bmatrix} &= \begin{bmatrix} 1 \\ E(t) \end{bmatrix} q + \begin{bmatrix} 0 \\ -E(t) \end{bmatrix} q_0, \end{aligned} \tag{S7}$$

where q and p are the volume and pressure in the chamber, respectively, and q_0 is the dead volume. The time varying elastance, $E(t)$, is given by

$$E(t) = e(t)(E_A) + E_B, \tag{S8}$$

where $e(t)$ is the normalised time varying elastance, E_A is the maximum chamber elastance minus the minimum chamber elastance, and E_B is the minimum chamber elastance. This chamber model and the $e(t)$ function in particular is equivalent to the one used in Liang et al. (2009) and Blanco and Feijóo (2013). However, we have extended $e(t)$ to allow dynamic modification of contraction and relaxation periods. As the heart period is fixed to $1.0\,s$ in this study, this extension is left out. We have verified that our model for $e(t)$ is equivalent to that used by Liang et al. (2009) for the fixed period.

The equations for valve dynamics have been taken from Mynard et al. (2012), and are given by

$$\frac{dv_v}{dt} = \frac{-B_v v^2 + p_1 - p_2}{I_v} \tag{S9}$$

where v_v is the flow through the valve, p_1 is the upstream pressure, and p_2 is the downstream pressure. B_v and I_v are nonlinear functions of the effective valve area, which is modelled as a first order dynamics function of pressure that simulates the opening and closing of the valves. For more details on the valve model, see Mynard et al. (2012).

REFERENCES

Blanco, P. J. and Feijóo, R. A. (2013). A dimensionally-heterogeneous closed-loop model for the cardiovascular system and its applications. *Medical Engineering and Physics* 35, 652–667. doi:10.1016/j.medengphy.2012.07.011

Liang, F., Takagi, S., Himeno, R., and Liu, H. (2009). Multi-scale modeling of the human cardiovascular system with applications to aortic valvular and arterial stenoses. *Medical and Biological Engineering and Computing* 47, 743–755. doi:10.1007/s11517-009-0449-9

Mynard, J. P., Davidson, M. R., Penny, D. J., and Smolich, J. J. (2012). A simple, versatile valve model for use in lumped parameter and one-dimensional cardiovascular models. *International Journal for Numerical Methods in Biomedical Engineering* 28, 626–641. doi:10.1002/cnm.1466

Safaei, S., Blanco, P. J., Müller, L. O., Hellevik, L. R., and Hunter, P. J. (2018). Bond graph model of cerebral circulation: Toward clinically feasible systemic blood flow simulations. *Frontiers in Physiology* 9, 1–15. doi:10.3389/fphys.2018.00148

Vlachopoulos, C., O'Rourke, M., and Nichols, W. W. (2011). *McDonald's blood flow in arteries: theoretical, experimental and clinical principles* (CRC press)

Waite, L. and Fine, J. (2007). *Applied Biofluid Mechanics* (McGraw-Hill Education)

Watanabe, S. M., Blanco, P. J., and Feijoó, R. A. (2013). Mathematical model of blood flow in an anatomically detailed arterial network of the arm. *ESAIM: Mathematical Modelling and Numerical Analysis* 47, 961–985. doi:10.1051/m2an/2012053

Westerhof, N. and Noordergraaf, A. (1970). Arterial viscoelasticity: A generalized model. Effect on input impedance and wave travel in the systematic tree. *Journal of Biomechanics* 3, 357–379. doi:10.1016/0021-9290(70)90036-9
