# Supplementary material for: Automated model calibration with parallel MCMC: Applications for a cardiovascular system model
Source: Front Physiol. 2022 Nov 9;13:1018134. doi: 10.3389/fphys.2022.1018134 (PMC9683692; doi:10.3389/fphys.2022.1018134)
Supplement: Supplementary file 2 [file DataSheet1.pdf]

Supplementary Material

1 SUPPLEMENTARY TABLES AND FIGURES

1.1 Patient 1

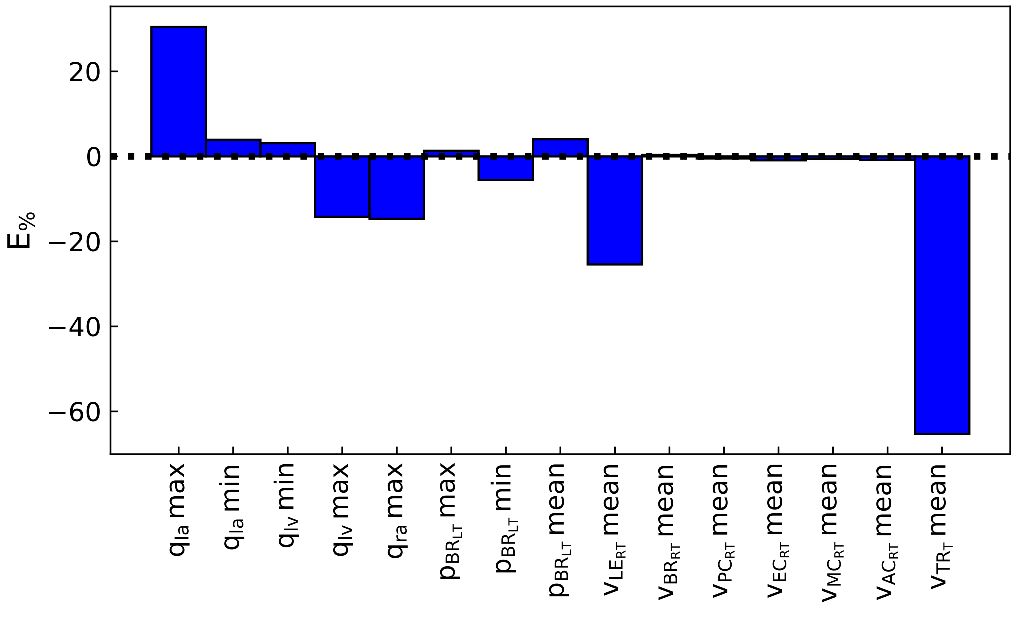

Figure S1. Percentage error between optimal model outputs ($f_i(\boldsymbol{\theta})$) and measured data ($\hat{z}_i$) from the parameter identification pipeline for patient 1 (normalised by $\hat{z}_i$). q_{la}: left atrium volume, q_{lv}: left ventricle volume, q_{ra}: right atrium volume, p_{BR_L}: left brachial pressure, $v_{LE_{RT}}$: right leg terminal flow, $v_{BR_{RT}}$: right brachial terminal flow, $v_{PC_{RT}}$: right posterior cerebral terminal flow, $v_{EC_{RT}}$: right external carotid terminal flow, $v_{MC_{RT}}$: right middle cerebral terminal flow, $v_{AC_{RT}}$: right anterior cerebral terminal flow, $v_{TR_{RT}}$: right trunk terminal flow

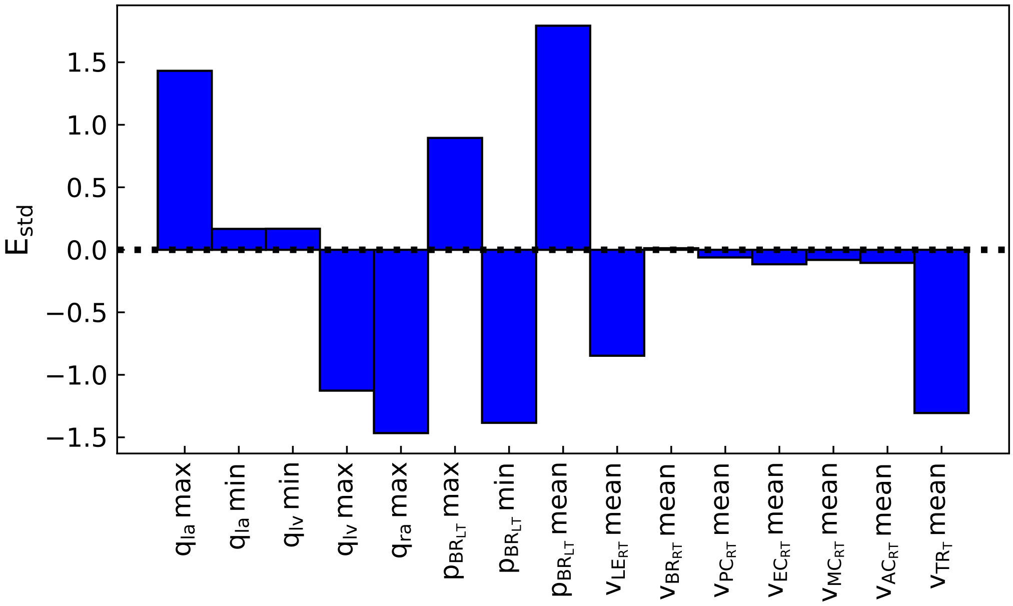

Figure S2. Normalised error between optimal model outputs ($f_i(\boldsymbol{\theta})$) and measured data ($\hat{z}_i$) from the parameter identification pipeline for patient 1 (normalised by σ_i). q_{la}: left atrium volume, q_{lv}: left ventricle volume, q_{ra}: right atrium volume, p_{BR_L}: left brachial pressure, $v_{LE_{RT}}$: right leg terminal flow, $v_{BR_{RT}}$: right brachial terminal flow, $v_{PC_{RT}}$: right posterior cerebral terminal flow, $v_{EC_{RT}}$: right external carotid terminal flow, $v_{MC_{RT}}$: right middle cerebral terminal flow, $v_{AC_{RT}}$: right anterior cerebral terminal flow, $v_{TR_{RT}}$: right trunk terminal flow

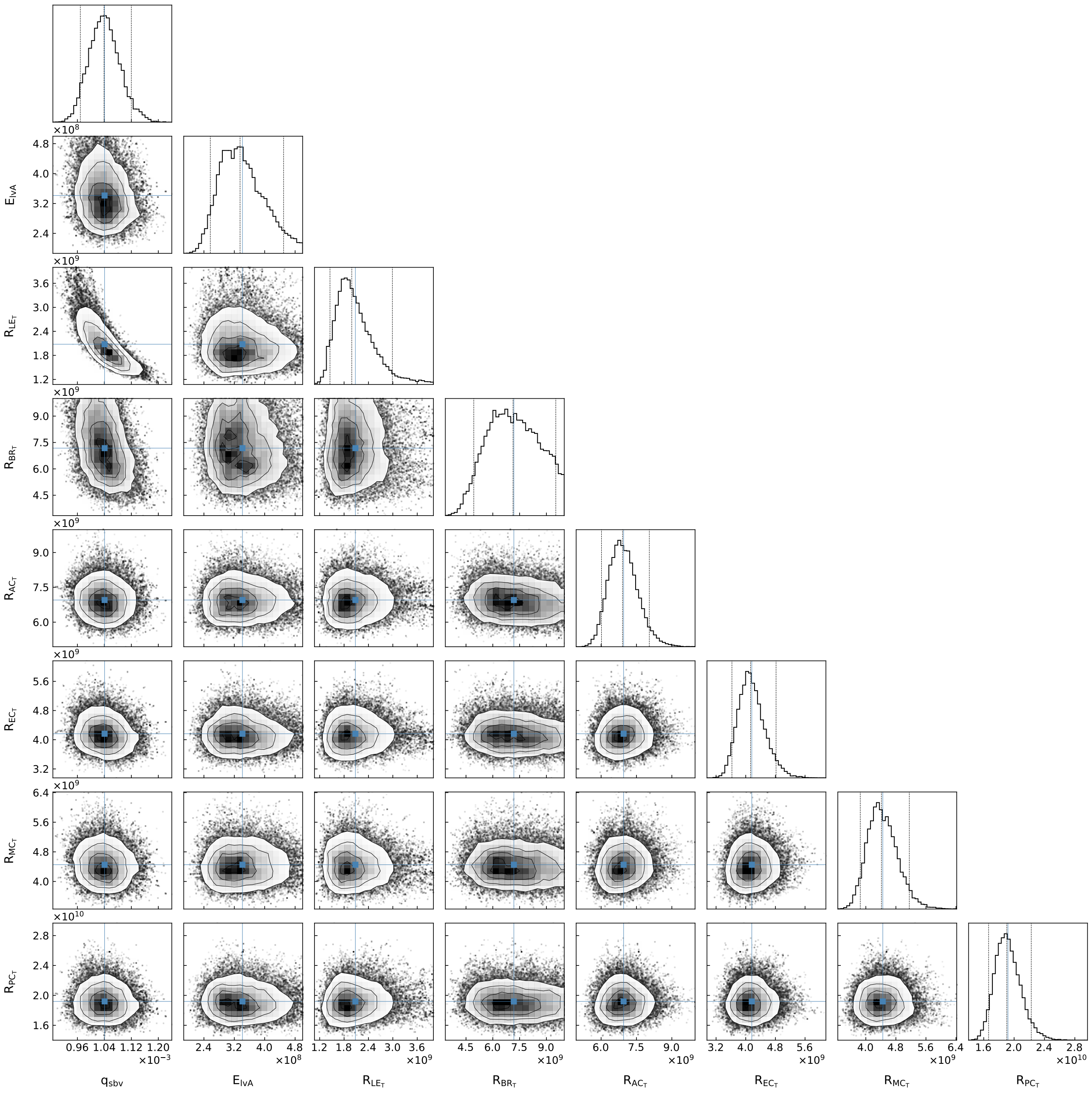

Figure S3. Parameter distributions for patient 1

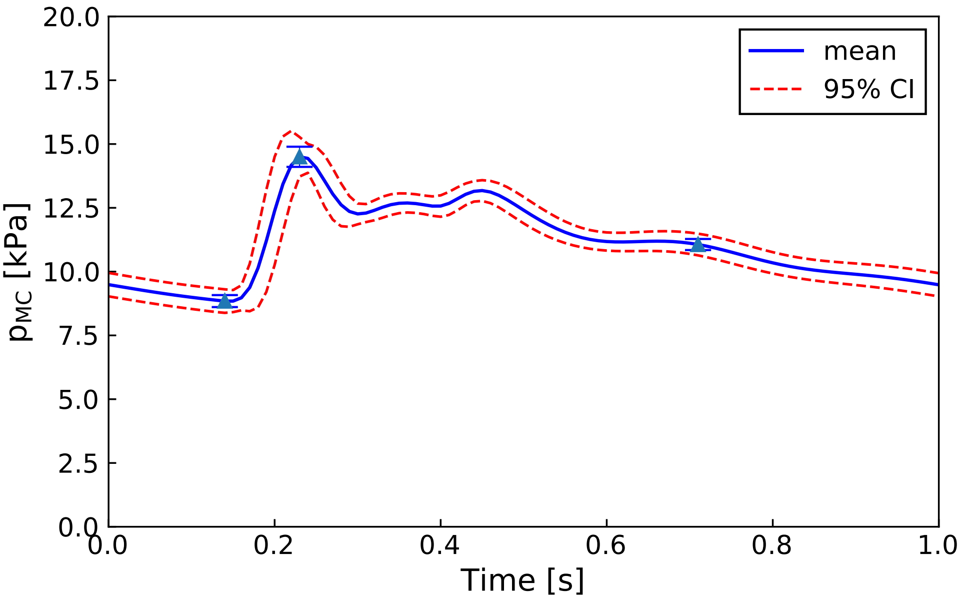

Figure S4. Patient 1: Mean, standard deviation, and 95% confidence interval for the middle cerebral artery pressure predictions (p_{MC}) sampled from the MCMC posterior parameter distributions (100 samples).

To compare the robustness of parameter fitting when specifying different prior distributions, we also ran the pipeline with a selection of normal and exponential prior distributions. The choice of priors is shown in Section 1.1 and the resulting posterior distributions after running the pipeline are shown in Figure S5. The normal distribution means were chosen to be the mean of the range between the max and min values for that parameter and the standard deviation was chosen to be $1/6$ times the range. These choices of the priors can be improved to provide improved physiological representation in future work. The current test was performed as a first test of robustness to non-uniform priors and shows that the resulting posterior distributions are very similar to the uniform prior case shown in Figure S3.

parameter	prior
q_{sbv}	normal
E_{LVa}	normal
R_{LE_T}	normal
R_{BR_T}	exponential
R_{AC_T}	exponential
R_{EC_T}	exponential
R_{MC_T}	exponential
R_{PC_T}	exponential

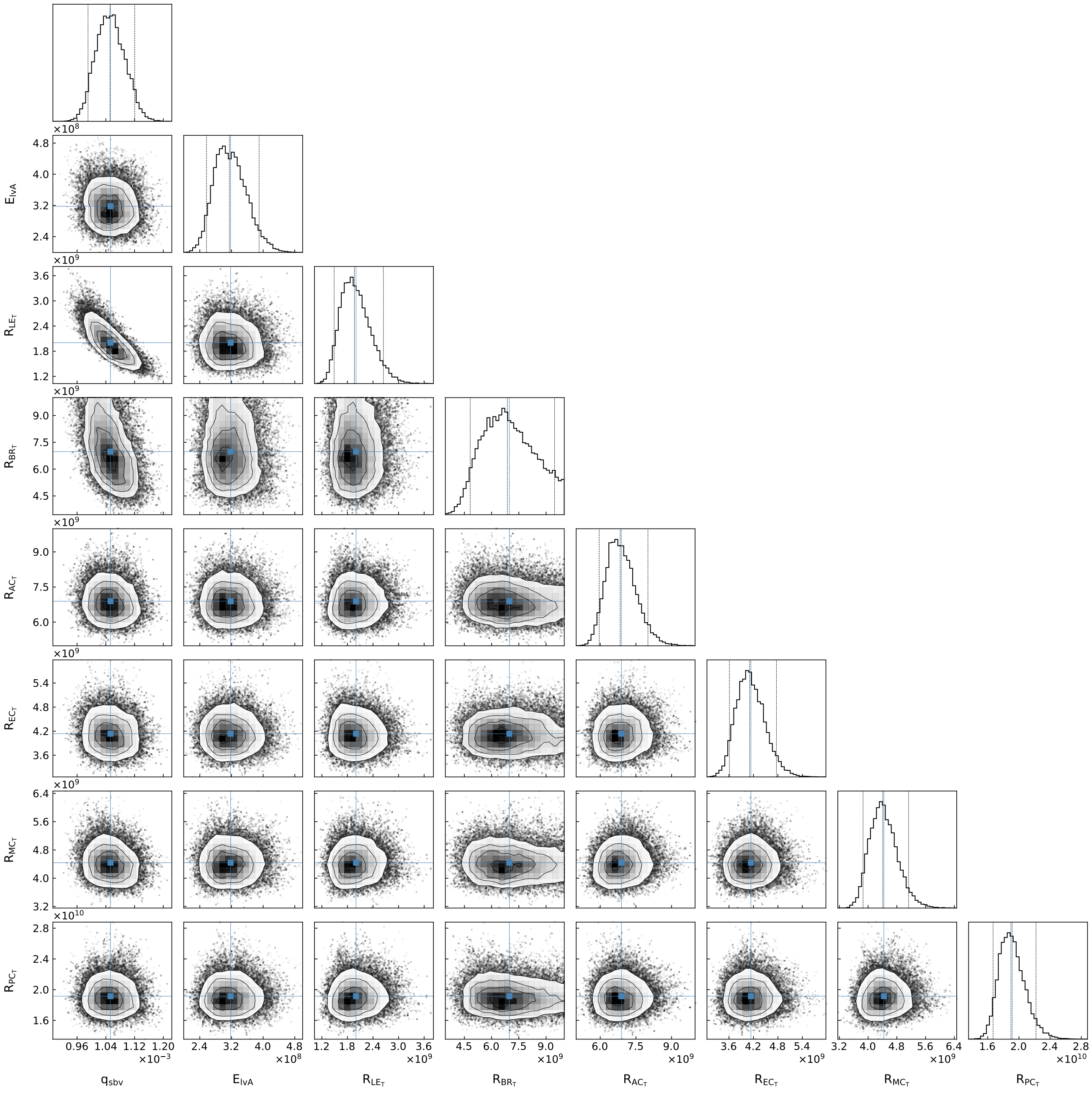

Figure S5. Parameter distributions for patient 1 with an assortment of normal and exponential parameter prior distributions.

1.2 Patient 2

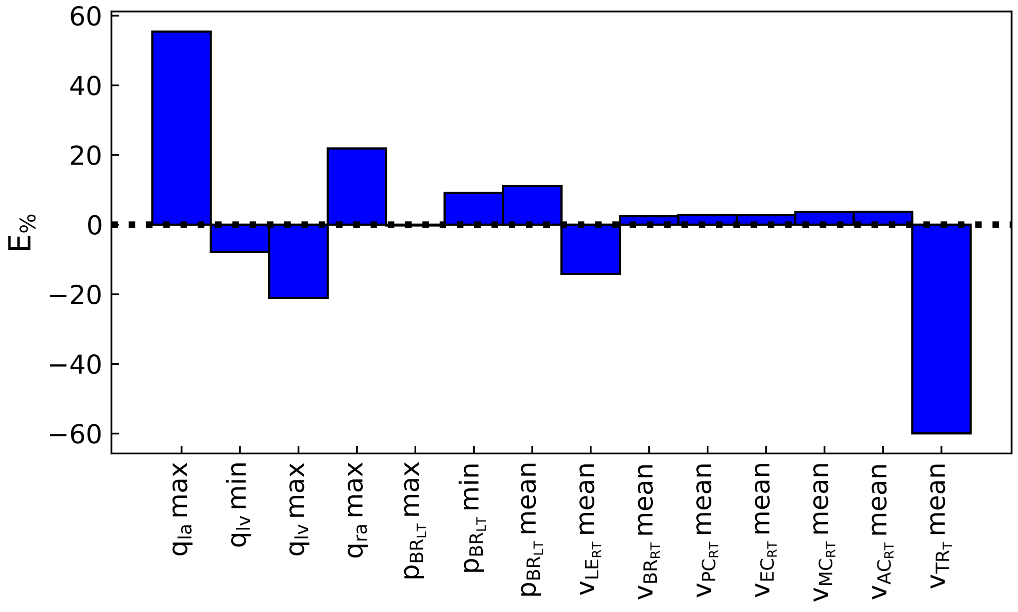

Figure S6. Percentage error between optimal model outputs ($f_i(\boldsymbol{\theta})$) and measured data ($\hat{z}_i$) from the parameter identification pipeline for patient 2 (normalised by $\hat{z}_i$). q_{la}: left atrium volume, q_{lv}: left ventricle volume, q_{ra}: right atrium volume, p_{BR_L}: left brachial pressure, $v_{LE_{RT}}$: right leg terminal flow, $v_{BR_{RT}}$: right brachial terminal flow, $v_{PC_{RT}}$: right posterior cerebral terminal flow, $v_{EC_{RT}}$: right external carotid terminal flow, $v_{MC_{RT}}$: right middle cerebral terminal flow, $v_{AC_{RT}}$: right anterior cerebral terminal flow, $v_{TR_{RT}}$: right trunk terminal flow

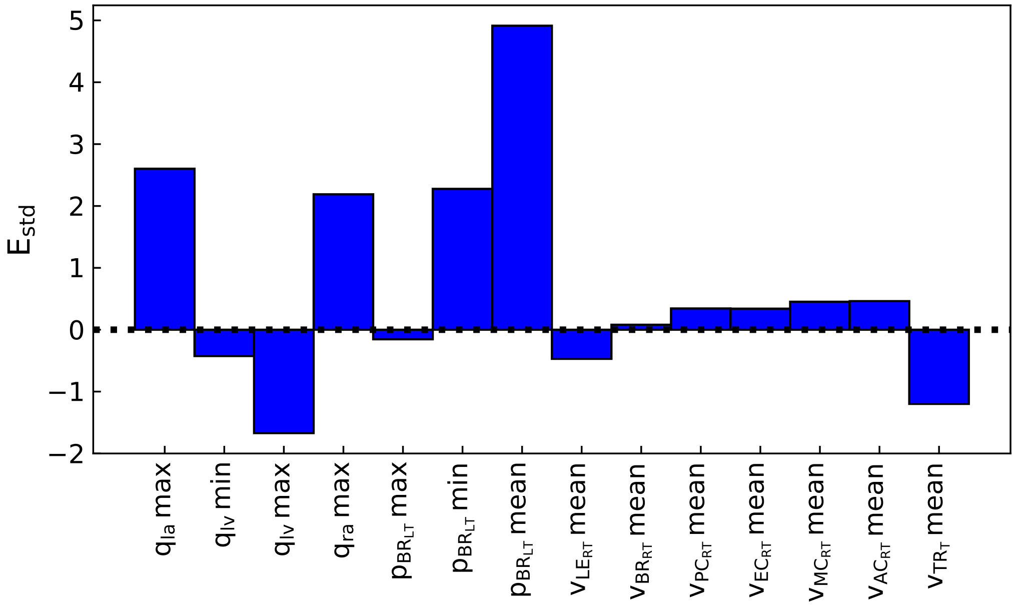

Figure S7. Normalised error between optimal model outputs ($f_i(\boldsymbol{\theta})$) and measured data ($\hat{z}_i$) from the parameter identification pipeline for patient 2 (normalised by σ_i). q_{la}: left atrium volume, q_{lv}: left ventricle volume, q_{ra}: right atrium volume, p_{BR_L}: left brachial pressure, $v_{LE_{RT}}$: right leg terminal flow, $v_{BR_{RT}}$: right brachial terminal flow, $v_{PC_{RT}}$: right posterior cerebral terminal flow, $v_{EC_{RT}}$: right external carotid terminal flow, $v_{MC_{RT}}$: right middle cerebral terminal flow, $v_{AC_{RT}}$: right anterior cerebral terminal flow, $v_{TR_{RT}}$: right trunk terminal flow

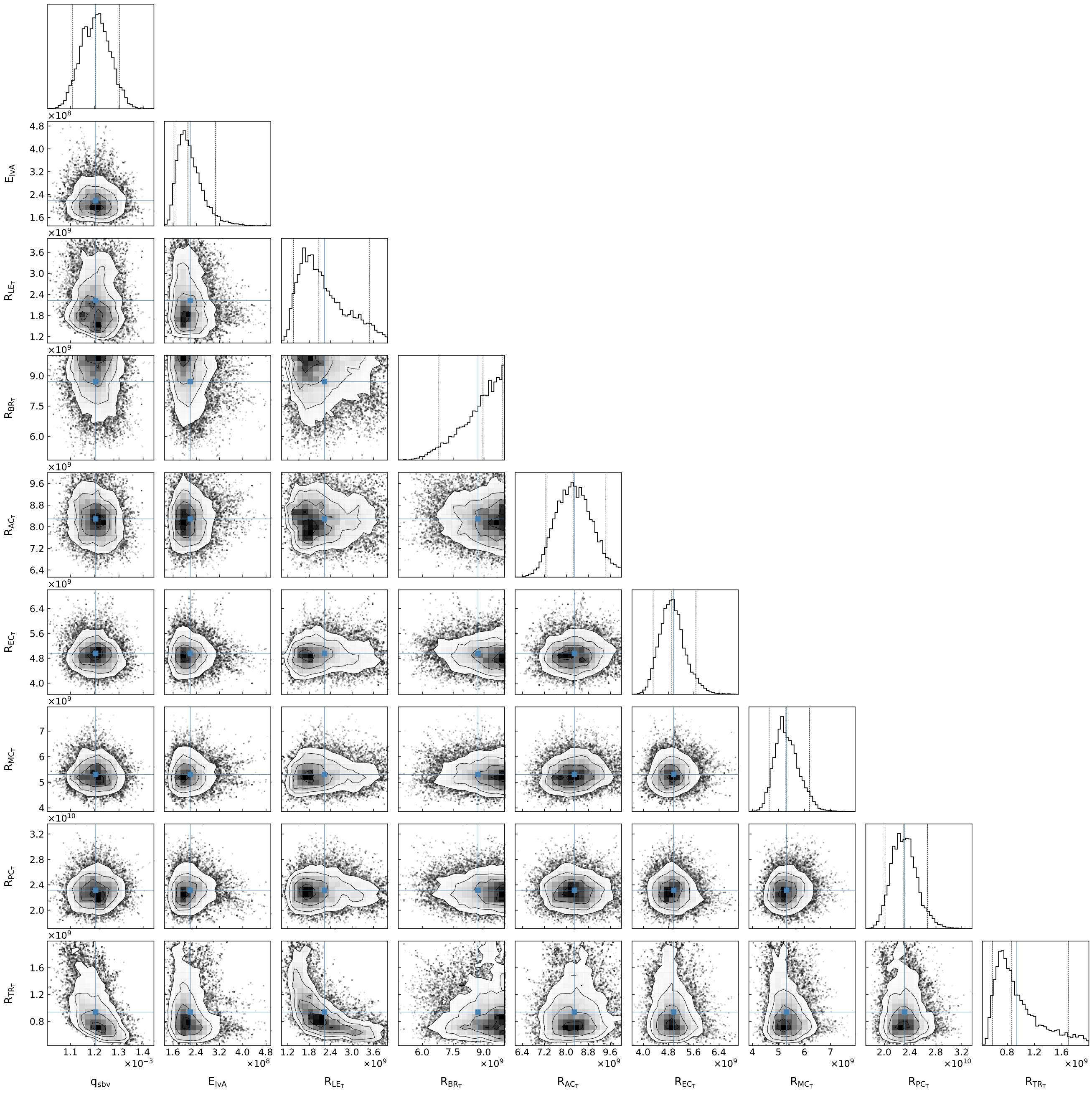

Figure S8. Parameter distributions for patient 2.

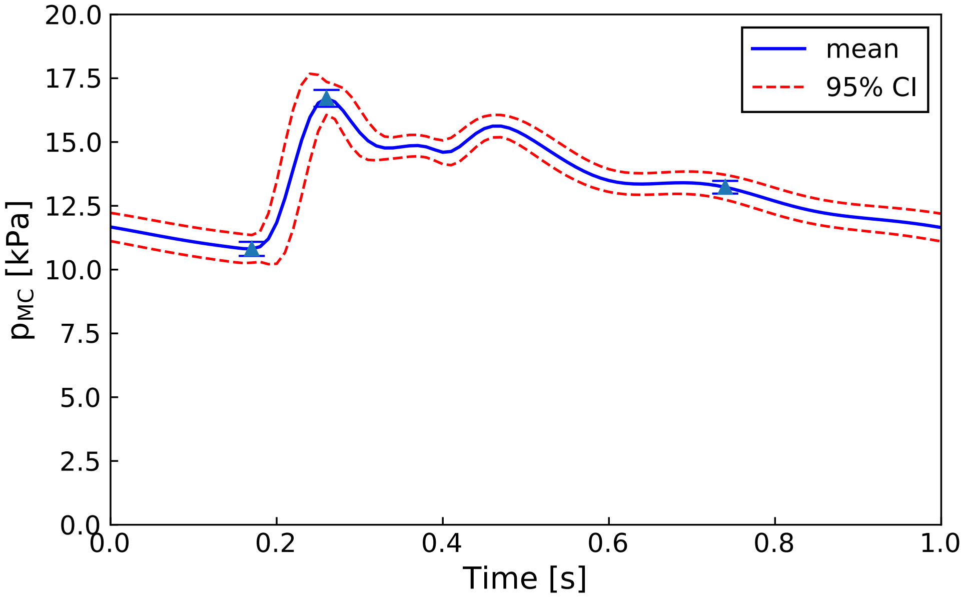

Figure S9. Patient 2: Mean, standard deviation, and 95% confidence interval for the middle cerebral artery pressure predictions (p_{MC}) sampled from the MCMC posterior parameter distributions (100 samples).

1.3 Patient 3

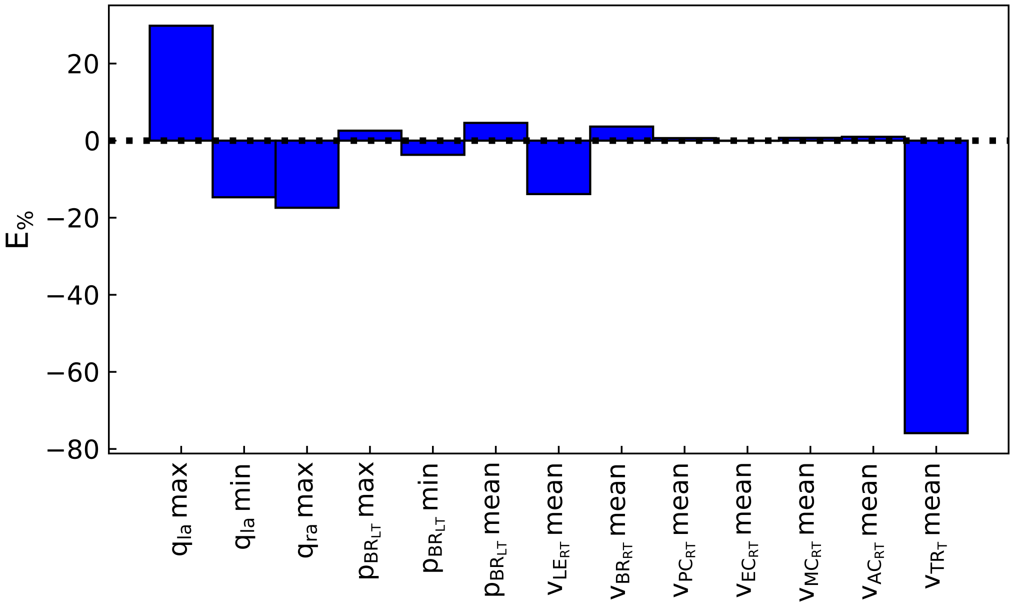

Figure S10. Percentage error between optimal model outputs ($f_i(\boldsymbol{\theta})$) and measured data ($\hat{z}_i$) from the parameter identification pipeline for patient 3 (normalised by $\hat{z}_i$). q_{la}: left atrium volume, q_{lv}: left ventricle volume, q_{ra}: right atrium volume, p_{BR_L}: left brachial pressure, $v_{LE_{RT}}$: right leg terminal flow, $v_{BR_{RT}}$: right brachial terminal flow, $v_{PC_{RT}}$: right posterior cerebral terminal flow, $v_{EC_{RT}}$: right external carotid terminal flow, $v_{MC_{RT}}$: right middle cerebral terminal flow, $v_{AC_{RT}}$: right anterior cerebral terminal flow, $v_{TR_{RT}}$: right trunk terminal flow

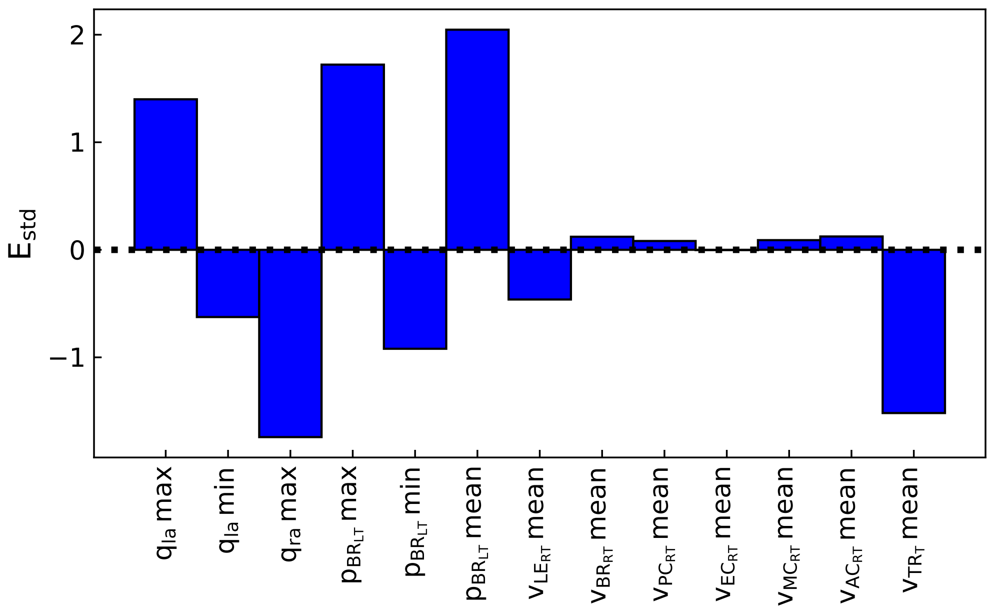

Figure S11. Normalised error between optimal model outputs ($f_i(\boldsymbol{\theta})$) and measured data ($\hat{z}_i$) from the parameter identification pipeline for patient 3 (normalised by σ_i). q_{la}: left atrium volume, q_{lv}: left ventricle volume, q_{ra}: right atrium volume, p_{BR_L}: left brachial pressure, $v_{LE_{RT}}$: right leg terminal flow, $v_{BR_{RT}}$: right brachial terminal flow, $v_{PC_{RT}}$: right posterior cerebral terminal flow, $v_{EC_{RT}}$: right external carotid terminal flow, $v_{MC_{RT}}$: right middle cerebral terminal flow, $v_{AC_{RT}}$: right anterior cerebral terminal flow, $v_{TR_{RT}}$: right trunk terminal flow

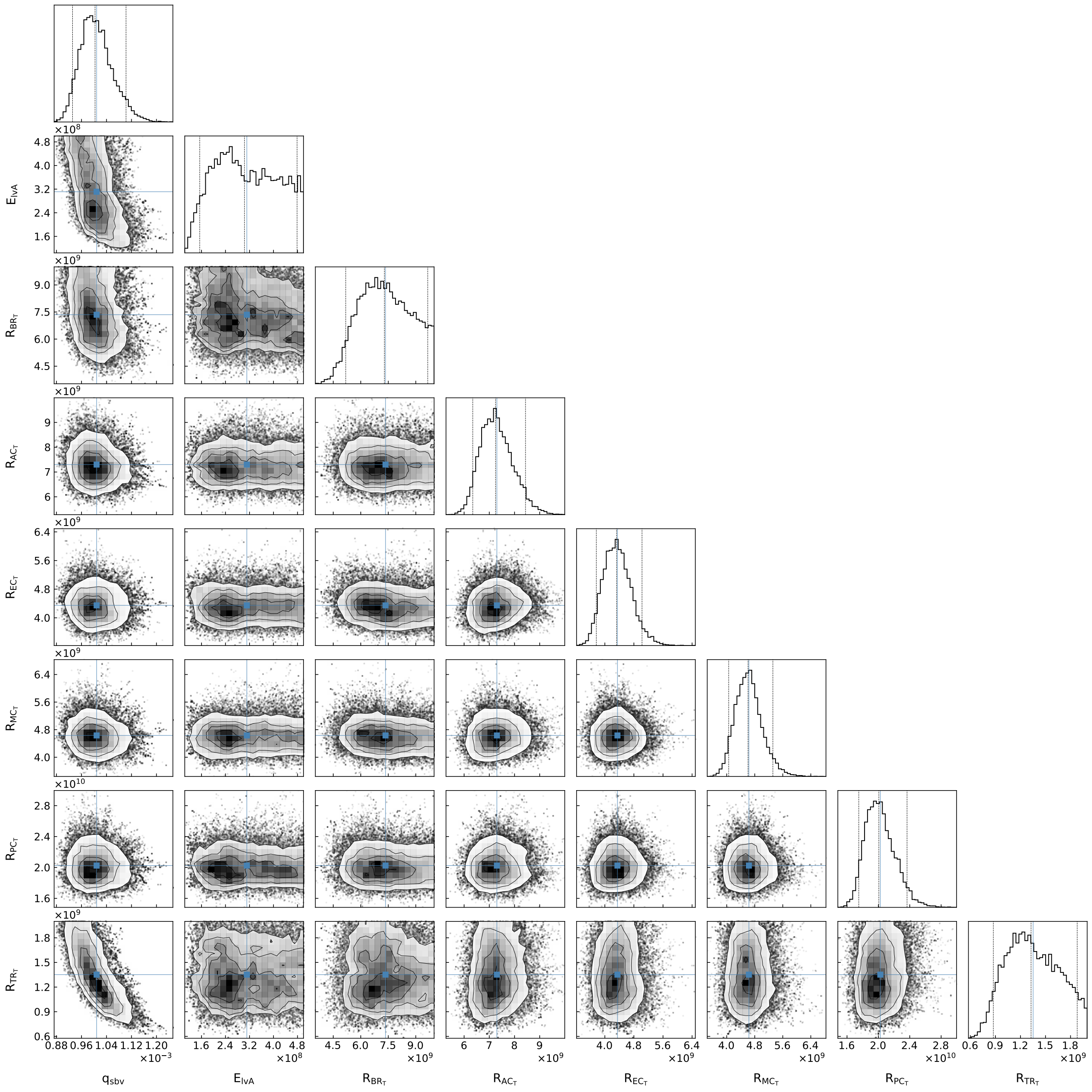

Figure S12. Parameter distributions for patient 3

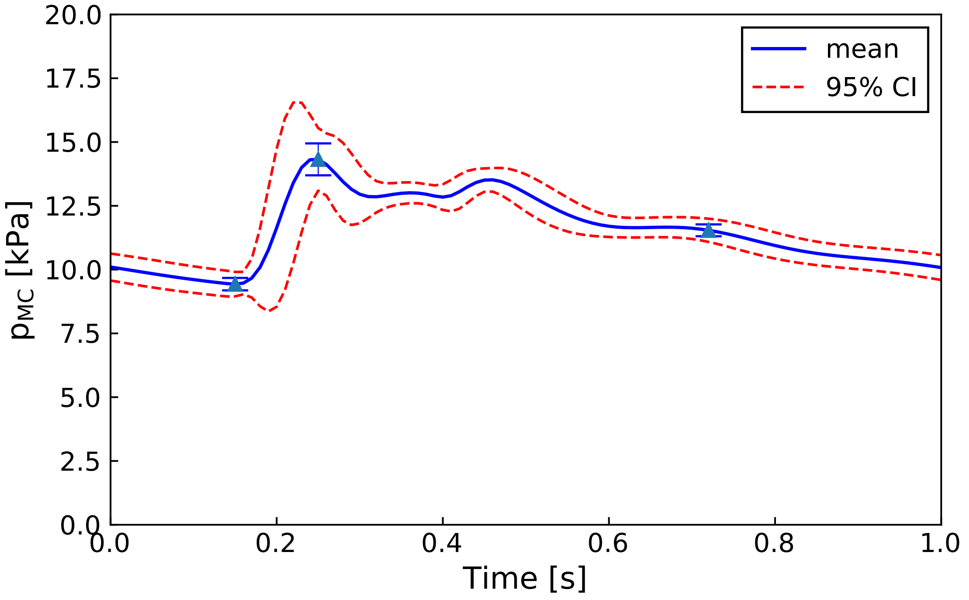

Figure S13. Patient 3: Mean, standard deviation, and 95% confidence interval for the middle cerebral artery pressure predictions (p_{MC}) sampled from the MCMC posterior parameter distributions (100 samples).
